# Possible epigenetic regulatory effect of dysregulated circular RNAs in epilepsy

**DOI:** 10.1371/journal.pone.0209829

**Published:** 2018-12-28

**Authors:** Woo-Jin Lee, Jangsup Moon, Daejong Jeon, Tae-Joon Kim, Jung-Suk Yoo, Dong-Kyu Park, Soon-Tae Lee, Keun-Hwa Jung, Kyung-Il Park, Ki-Young Jung, Manho Kim, Sang Kun Lee, Kon Chu

**Affiliations:** 1 Department of Neurology, Comprehensive Epilepsy Center, Laboratory for Neurotherapeutics, Biomedical Research Institute, Seoul National University Hospital, Seoul, South Korea; 2 Program in Neuroscience, Neuroscience Research Institute of SNUMRC, College of Medicine, Seoul National University, Seoul, South Korea; 3 Department of Neurology, Seoul National University Healthcare System Gangnam Center, Seoul, South Korea; University of Modena and Reggio Emilia, ITALY

## Abstract

Circular RNAs (circRNAs) involve in the epigenetic regulation and its major mechanism is the sequestration of the target micro RNAs (miRNAs). We hypothesized that circRNAs might be related with the pathophysiology of chronic epilepsy and evaluated the altered circRNA expressions and their possible regulatory effects on their target miRNAs and mRNAs in a mouse epilepsy model. The circRNA expression profile in the hippocampus of the pilocarpine mice was analyzed and compared with control. The correlation between the expression of miRNA binding sites (miRNA response elements, MRE) in the dysregulated circRNAs and the expression of their target miRNAs was evaluated. As miRNAs also inhibit their target mRNAs, circRNA–miRNA-mRNA regulatory network, comprised of dysregulated RNAs that targets one another were searched. For the identified networks, bioinformatics analyses were performed. As the result, Forty-three circRNAs were dysregulated in the hippocampus (up-regulated, 26; down-regulated, 17). The change in the expression of MRE in those circRNAs negatively correlated with the change in the relevant target miRNA expression (r = -0.461, P<0.001), supporting that circRNAs inhibit their target miRNA. 333 dysregulated circRNA–miRNA-mRNA networks were identified. Gene ontology and pathway analyses demonstrated that the up-regulated mRNAs in those networks were closely related to the major processes in epilepsy. Among them, STRING analysis identified 37 key mRNAs with abundant (≥4) interactions with other dysregulated target mRNAs. The dysregulation of the circRNAs which had multiple interactions with key mRNAs were validated by PCR. We concluded that dysregulated circRNAs might have a pathophysiologic role in chronic epilepsy by regulating multiple disease relevant mRNAs via circRNA−miRNA−mRNA interactions.

## Introduction

Epilepsy is a chronic disease resulting from a long-term process of epileptogenesis in the brain.[1–3] Regardless of the type of primary insult, common reactive processes occur around the injured brain tissue during the latent period, forming an aberrant neural network that generates synchronous hyper excitation of neurons, which manifests as spontaneous recurrent seizures.[1–5] In this regard, most of the patients with epilepsy have little chance to be treated during the period of epileptogenesis, and the treatment usually starts when the seizure events have already been evident. Therefore, elucidating the detailed genetic regulation in the brain with chronic epilepsy and finding strategies to modulate it might be fundamental to improve the efficacy of the chronic epilepsy treatment.

The genes involved in epileptogenesis are tightly regulated by various epigenetic regulatory mechanisms, and their chronic alteration in the hippocampus might have a major role of temporal lobe epilepsy.[2–4, 6] Among them, micro RNAs (miRNAs), small (20–24 bp), noncoding RNAs (ncRNAs) that regulate the expression of hundreds of target genes, have been most widely investigated.[7, 8] However, recent evidence has demonstrated that gene expression is more precisely modulated by another type of ncRNA, called circular RNA (circRNA).[9–12]

CircRNAs are a covalently closed and circular-shaped subgroup of ncRNAs.[9, 10] Predominantly, circRNAs are generated by back-splicing, a process by which downstream exons are reversely spliced to upstream exons.[9, 10] circRNAs are increasingly recognized as major epigenetic regulators in the pathogenesis of various diseases.[9–14] A circRNA might interact with the gene transcription machineries and the production of a circRNA might complete with the production of its corresponding linear form mRNA.[15] Moreover, circRNAs contains miRNA binding sites, the miRNA response elements (MREs), that enable circRNAs to sequestrate the target miRNA, which is known as the “miRNA sponge effect”.[9, 10] In this regard, circRNAs might modulate the expression of their target genes via circRNA-miRNA-mRNA regulatory networks.

Some properties of circRNAs indicate that circRNAs might have a particular role in the pathomechanism of the central nervous system (CNS) diseases. First, circRNAs are highly abundant in CNS and their expression level is tightly regulated according to the time and the region in the brain.[16] Second, because circRNAs have covalently closed loop structures without polyA tails, they are more stable in the CNS by being resistant to RNA exonucleases or RNase R-mediated degradation.[9, 11] Therefore, studying the alteration of circRNA expression and its implication on the balance between the downstream target miRNAs and mRNAs might shed light on the understanding of pathophysiologic role of circRNAs in chronic epilepsy and provide a novel therapeutic target.[9–11]

In this study, we evaluated the comprehensive profile of differentially regulated circRNAs in the hippocampus of pilocarpine epilepsy model and tried to identify their functions in epigenetic regulatory processes involved in the pathophysiology of chronic epilepsy.

## Materials and methods

### Tissue preparation

Our study group have been generating pilocarpine chronic epilepsy models for several years.[6, 17–23] The epilepsy model used in the current study was randomly selected from a large group of pilocarpine mouse models in our laboratory, generated according to the previously described procedures.[6, 17–23] In brief, a single intraperitoneal injection of pilocarpine (330–400 mg/kg; Sigma, St. Louis, MO, USA) was performed in 118 male C57BL/6 mice to induce status epilepticus (SE). The age of the mice was set as 5 weeks, based on our laboratory experience that the frequency of developing spontaneous recurrent seizures (SRSs) was the highest at this age.[17, 18, 20–22] Methyl-scopolamine (1 mg/kg; Sigma) was intraperitoneally administered 30 min before the pilocarpine injection to minimize muscarinic adverse effects. 86.6% (102/118 mice) developed status epilepticus (SE) and at about 40 minutes after the onset of SE, intraperitoneal diazepam (5 mg/kg) was administered to convert the form of SE from convulsive to non-convulsive.[24] 41.5% (49 mice) died during or shortly after the procedure. Sixty days after SE, all of the fifty-three mice that survived after the prolonged SE developed clinical SRSs, which is consistent with the previous reports that demonstrated a 100% frequency of developing SRSs after inducing a prolonged SE by pilocarpine injection in rodents.[25–27] 24/7 continuous video-electroencephalograph (EEG) monitoring was performed in randomly selected twenty-seven mice for the mean duration of 53.7±20.4 days, and all of the monitored mice were confirmed to have SRSs with the mean seizure frequency of 2.0±0.6/day. As the video-EEG monitoring requires craniotomy and insertion of electronic probes into cerebral hemispheres which might effect as a trauma and induce a significant alteration in the RNA expression profiles in the brain, [28, 29] four mice used in the current study were randomly selected from the remaining 26 mice that were developed SE after pilocarpine injection, but did not underwent video-EEG monitoring. To compare the expression profiles of the circRNAs in the hippocampus between the pilocarpine chronic epilepsy model and controls, four age and sex-matched mice were also allocated to the control group.

Mice were euthanized by cervical dislocation and brains were immediately removed.[6, 17–23] The hippocampus was obtained from each mouse and were immediately stored at −80°C. All animals were managed with standardized procedures approved by the Institutional Animal Care and Use Committee of Seoul National University Hospital. All procedures were approved by the institutional review boards of the Seoul National University Hospital.

### Microarray

Total RNAs were extracted using TRIZOL reagent (Invitrogen, NY, USA) and purified by an RNeasy Mini Kit (Qiagen, Hilden, Germany). RNA quantity was measured with a Nanodrop ND-1000 (Thermo Fisher Scientific, MA, USA) and quality checked by an Agilent 2100 Bioanalyzer (Agilent Technologies, CA, USA). Sample preparation and microarray hybridization were performed according to the manufacturer’s protocol (Arraystar, Rockville, MD, USA). For circRNA microarray, the RNAs were treated with RNase R (Epicenter, WI, USA) to remove linear RNAs and enrich circRNA. The enriched circRNA samples were amplified and transcribed into fluorescent cRNA using a random priming method (Arraystar Super RNA Labeling Kit). The labeled cRNAs were hybridized onto the Arraystar Mouse circRNA Array V2 (8 × 15K, Arraystar). After the slides were washed, the arrays were scanned by an Agilent Scanner G2505C (Agilent Technologies). The miRNA and mRNA microarray data were also obtained using the Agilent Mouse miRNA Microarray 8X15K kit and Agilent Mouse Gene Expression Microarray 4X44K kit respectively, according to the manufacturer’s protocol (Agilent Technologies).

Agilent Feature Extraction software (version 11.0.1.1, Agilent Technologies) was used to analyze the acquired array images. Quantile normalization and subsequent data processing were performed with the R software limma package (R version 3.2.4). Low-intensity filtering was performed and RNAs with two or more out of eight samples that had flags in “P” or “M” were included in further analyses. Mann–Whitney test were used to detect differentially expressed circRNAs between the two groups by fold-changes of ≥1.5 and *P*-values ≤0.05.[17, 20, 21, 23] Volcano plot filtering and hierarchical clustering were also performed to identify differentially expressed circRNAs. In the analyses of miRNA and mRNA microarray, an false discovery rate (FDR) adjusted *P*-value of <0.05 with fold-changes of ≥1.5 was used to detect differentially expressed RNAs between the two groups.

### Analysis of the regulative effect of circRNA on its linear mRNA expression

To examine whether the dysregulated circRNAs regulate the expression of their linear form mRNAs, the expression ratio of each circRNA between the epilepsy and the control groups (**circRNA expression ratio**) were compared with the expression ratio of its linear form mRNAs (**mRNA expression ratio**).[30] Pearson’s correlation analysis was performed to measure the correlations between the expression ratios of circRNA and its linear form mRNAs.

### Analysis of the regulative effect of circRNA on target miRNA expression

**Fig 1:**
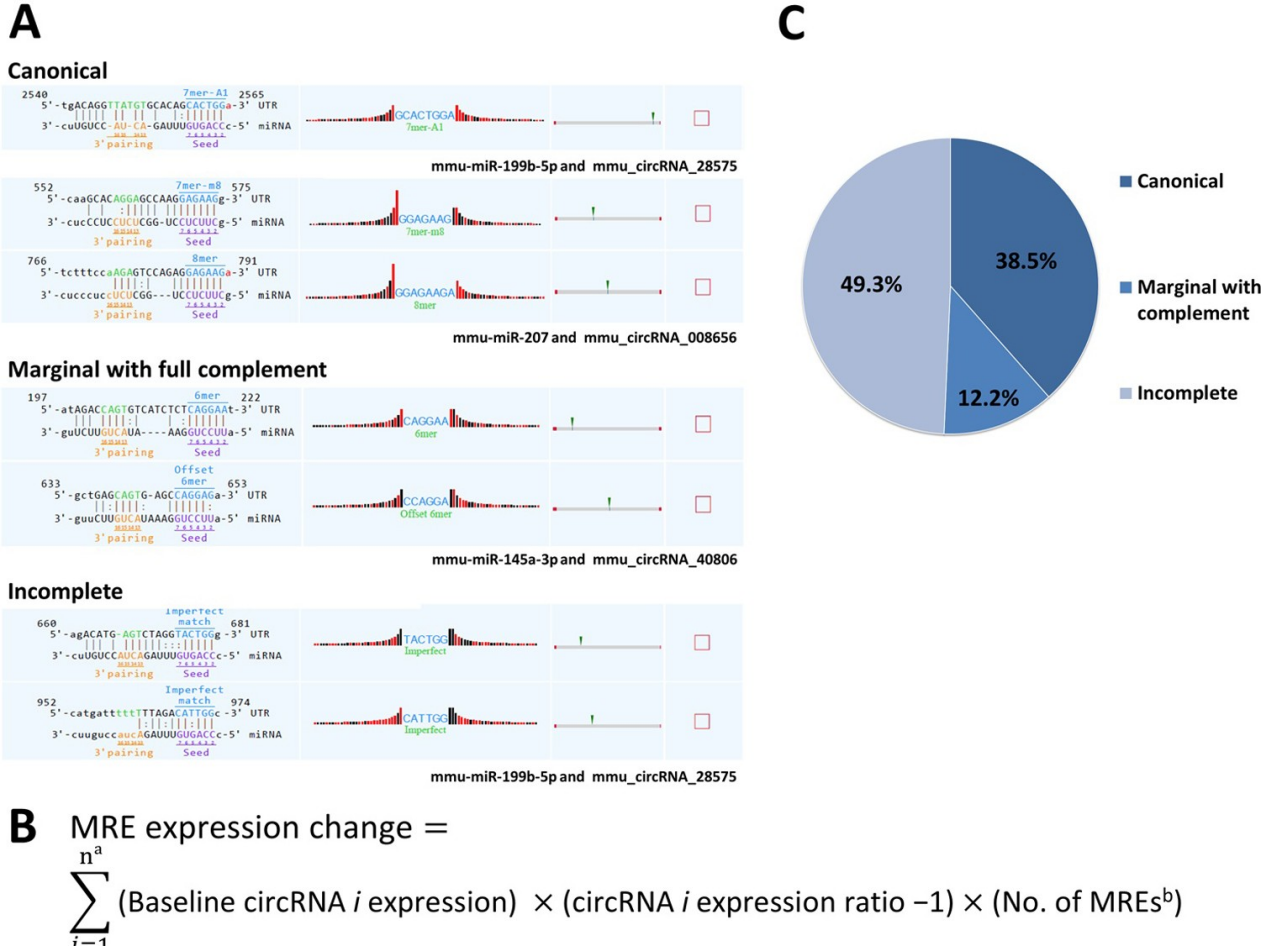
Analysis of the regulative effect of circRNA on miRNA expression. Each predicted MRE was composed of “seed site” and “complementary sites”. MREs were categorized into canonical, marginal with full complement, and incomplete, where a canonical denotes a seed site with 7–8 matching nucleotides, marginal with full complement denotes a seed site with 6 matching nucleotide with a complementary site with ≥4 canonical matching nucleotides, and incomplete denotes a seed site with 5–6 matching nucleotide or a complementary site with <4 canonical matching nucleotides (panel A). Panel B demonstrates how the relative value of MRE expression change in dysregulated circRNAs was calculated. Panel C denotes the proportion of each MRE categories. a: When two or more dysregulated circRNAs had MREs targeting a common miRNA, their change in the expression of MREs were summated. b: Calculated separately for each categories of MRE. MRE, miRNA response element.

To evaluate the hypothesis that circRNAs function as miRNA sponges, we tried to quantitatively analyze the correlation between the change in the expression of target miRNA binding sites in dysregulated circRNAs and the change in the expression of their target miRNAs (**target miRNA expression ratio**) according to the following steps. First, for every differentially expressed circRNA, up to five target miRNAs were identified by MRE sequence analysis.[31] MRE sequence analysis was performed using a miRNA target prediction software (Arraystar) based on TargetScan (www.targetscan.org) and miRanda (http://www.microrna.org) algorithms. Each MRE was composed of “seed site” and “complementary sites”. A seed site was designated as the ≥6 consecutive nucleotides matching to the miRNA nucleotides 2–7 from the 5′-end, whereas a complementary site was defined as the neighboring ≥4 sequences pairing to miRNA nucleotides 12–19.[32] Second, each MREs were categorized into canonical, marginal with full complement, and incomplete, where a canonical denotes a seed site with ≥7 consecutive matching nucleotides (7mer+A1, 7mer+m8, 8mer), marginal with full complement denotes a seed site with 6 matching nucleotide and a complementary site with ≥4 consecutive matching nucleotides, and incomplete denotes a seed site with <6 matching nucleotide and a complementary site with <4 matching nucleotides.[32] A circRNA could contain multiple MREs for a given target miRNA and vice versa. Third, the change in the expression of MRE was calculated for each MRE categories, by multiplying the normalized baseline expression intensity of the corresponding circRNA, circRNA expression ratio -1, and the number of MREs of the relevant category for the target miRNA. When two or more dysregulated circRNAs were targeting a common miRNA, their changes in the expression of MREs were summated to evaluate the comprehensive effect of multiple circRNAs on a given target miRNA (**Fig 1A and 1B**). Finally, correlations between the change in the expression of MRE and the target miRNA expression ratio were measured, separately in each MRE categories.

### Specific circRNA–miRNA–mRNA regulatory network

In order to search the possible inhibitory interaction between the differentially expressed circRNAs and their target miRNAs, significantly down-regulated downstream miRNAs for the up-regulated circRNAs or significantly up-regulated downstream miRNAs for the down-regulated circRNAs were designated as circRNA-interacting miRNAs (CI-miRNAs) and were included in further analyses. Then, to predict the target mRNAs regulated by the CI-miRNAs, the up- and down-regulated CI-miRNA sets were separately entered into an integrative miRNA target prediction program miRsystem (http://mirsystem.cgm.ntu.edu.tw), along with their expression ratio data.[33] After the list of potential target mRNAs was obtained, their expression ratios were also extracted from the mRNA microarray data. Significantly down-regulated downstream target mRNAs for the up-regulated CI-miRNA sets or significantly up-regulated downstream target mRNAs for the down-regulated CI-miRNA sets were defined as circRNA-miRNA-interacting mRNAs (CMI-mRNAs).

### Gene ontology and pathway analysis

To demonstrate the pathophysiologic role of the circRNA–miRNA–mRNA regulatory network in chronic epilepsy, gene ontology and pathway analyses were performed for the CMI-mRNAs. The gene ontology categories were obtained from the Gene Ontology website (http://www.geneontology.org).[23] Pathway analysis was performed using the Kyoto Encyclopedia of Genes and Genomes (http://www.genome.jp/kegg) database.[23, 34] In both analyses, categories with *P* < 0.05 were considered to be statistically significant.

### STRING analysis

Interaction among the proteins translated by CMI-mRNAs was analyzed with STRING v10 (http://string-db.org). A network map was drawn by analyzing a protein–protein interaction database derived from multiple sources as follows: primary experimental databases, biological pathway databases, automated text-mining from Medline abstracts and a large collection of full-text articles, and de novo interactions predicted by genomic information algorithms.[35] The minimum required score for determining a significant interaction was set as 0.400, corresponding to a medium confidence.[35]

### Quantitative PCR analysis

Five differentially expressed circRNAs (one up-regulated, four down-regulated) were selected for quantitative real-time reverse transcription PCR analysis to validate the microarray expression data. For PCR analysis, two hippocampal tissue samples were pooled into one RNA sample as a unit. cDNAs were synthesized from 0.5 μg of total RNA of hippocampal tissues by reverse transcription. Standard curves were prepared using 2× SuperArray PCR master mix (Arraystar) according to the manufacturer’s protocol. The relative expression ratio of each circRNA was calculated with the Rotor-Gene Real-Time Analysis Software 6.0 (Qiagen), using the housekeeping gene, *Gapdh*, expression for normalization. All real-time reactions were performed in triplicate.

### Statistical analysis

Data were reported as number (percentage) or mean ± standard deviation. Excel 2016 (Microsoft, Redmond, WA, USA) was used for the Mann–Whitney *U* test to detect differentially expressed circRNAs, miRNAs, and mRNAs between the two groups by fold-changes of ≥1.5 and *P*-values ≤0.05.[17, 20, 21, 23] Correction for the multiple comparisons were not performed for the circRNA microarray analysis, due to that the portion of dysregulated circRNAs in microarray analysis was too small to perform the Benjamini-Hochberg procedure for p-value adjustment. In the analyses of miRNA and mRNA microarray, Benjamini-Hochberg procedure was performed to control the FDR at 0.05, and an adjusted *P*-value of <0.05 was used to detect differentially expressed RNAs between the two groups.

SPSS (version 22.0; SPSS Inc., Chicago, IL, USA) was used to perform Pearson’s correlation analyses to measure the correlations between the expression ratios of the differentially expressed circRNAs and their linear form mRNAs and the correlations between the change in the expression of MRE and the target miRNA expression ratio. *P* values < 0.05 were considered statistically significant.

## Results

### Overall expression profile of circRNA

**Fig 2:**
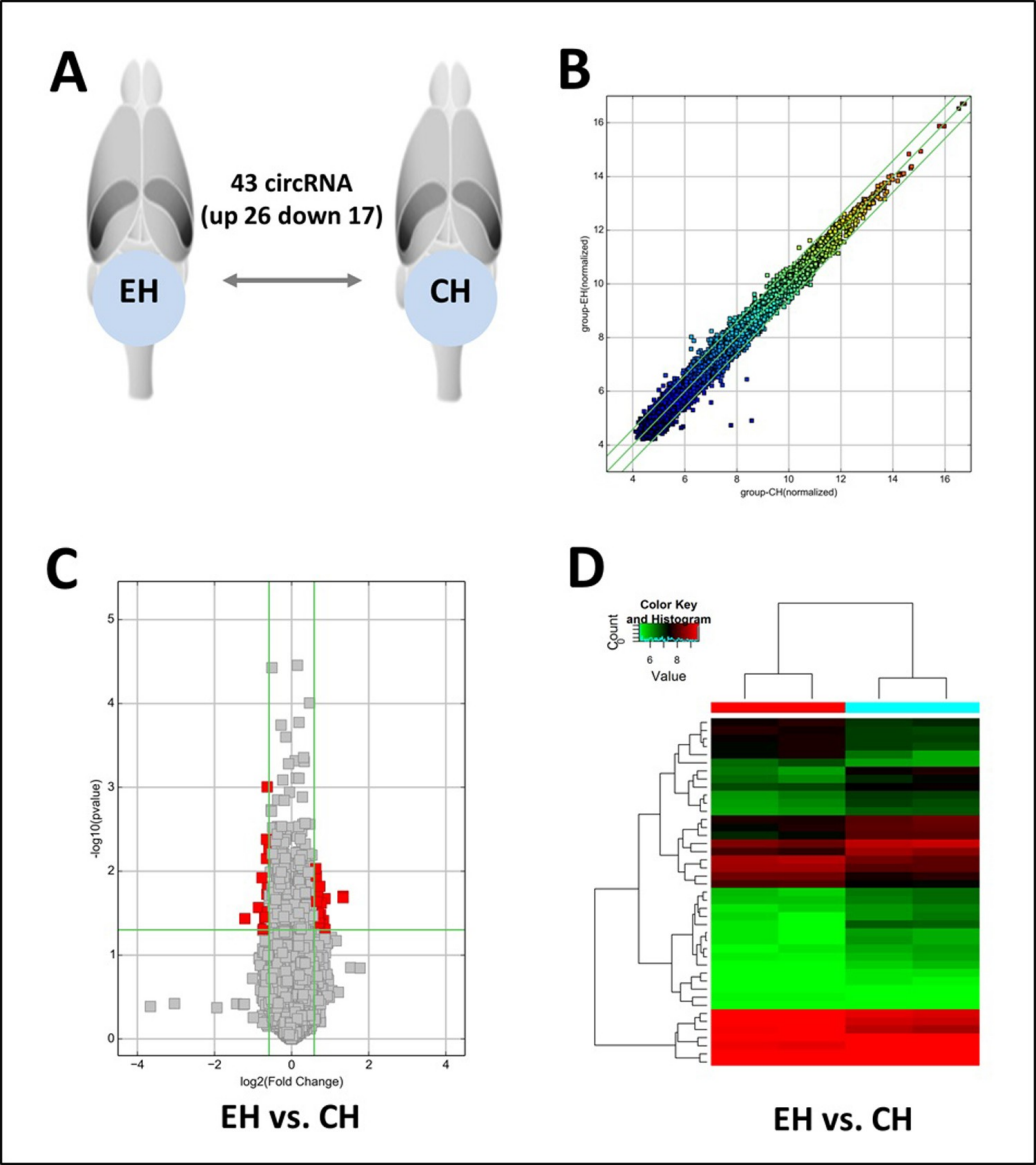
Microarray data analysis of the differentially regulated circRNAs in the hippocampus of the pilocarpine epilepsy model. The numbers of circRNAs that are differentially expressed between the hippocampi of models are shown in panel A. Scatter Plot (panel B) shows that for most circRNAs, their expressions in the epilepsy model and the control were comparable. Volcano plot shows differentially expressed circRNAs in the hippocampus of the pilocarpine epilepsy model by red squares (panel C). The left and right vertical lines demark two-fold up and two-fold down changes, respectively, whereas the horizontal line indicates a *P*-value of 0.05. Panel D shows hierarchical cluster analysis of differentially expressed circRNAs in the hippocampus of the pilocarpine epilepsy and control mice. The log2 signal intensity is reflected in the color scale, which runs from blue (low intensity) to red (strong intensity).

Among a total of 12,984 circRNAs analyzed, the number of differentially expressed circRNAs in the hippocampus of pilocarpine model was 43 (up-regulated, 26; down-regulated, 17, **Fig 2**). The differentially expressed circRNAs are listed with their expression ratios in the **S1 Table** (Full circRNA expression profile in **S1 File**).

### circRNA expression is not associated with their linear mRNA expression

The expression ratios of 10,607 linear form mRNAs out of 12,984 circRNAs (89.3%) were available. Among them, 598 (5.6%) mRNAs were significantly upregulated and 71 (0.7%) mRNAs were significantly downregulated. However, the expression ratio of the circRNAs did not correlate with the expression ratio of their linear form mRNAs (*r* = 0.002, *P* = 0.886).

### Binding site expression in dysregulated circRNA is negatively associated with their target miRNA expression

The MRE analysis identified 215 potential interactions between the 43 dysregulated circRNA and their downstream target miRNAs (five miRNAs for each dysregulated circRNA). Among them, the target miRNA expression ratio was available in 75 interactions (34.9%) from the microarray data (full data in **S2 File**). The total number of MREs in those 75 circRNA-miRNA interactions was 286 (3.81±5.60, range 1−46 MREs per one interaction), consisting of 110 (38.5%) canonical type, 35 (12.2%) marginal with full complement type, and 141 (49.3%) incomplete type MREs (**Fig 1C**).

In the correlation analyses between the change in the expression of MRE in dysregulated circRNAs and the target miRNA expression ratio, the change in the all-type MRE expression negatively correlated with the target miRNA expression ratio (*r* = -0.249, *P* = 0.032). The association was higher for the canonical or marginal with full complement types of MRE (*r* = -0.515, *P*<0.001 and *r* = -0.461, *P*<0.001, respectively), whereas no significant association was found between the incomplete type MRE and the target miRNA (*r* = -0.097, *P* = 0.407, **Table 1**).

**Table 1:** Correlation analyses between the MRE change amount and the target miRNA expression ratio.

circRNA categories		MRE categories			
	All-type (n = 286)	Canonical (n = 110)	Marginal with full complement (n = 35)	Incomplete (n = 141)
**All-type** (n = 75)	*r* = -0.249 P = 0.032	*r* = -0.515 P < 0.001	*r* = -0.355 P = 0.002	*r* = -0.097 P = 0.407
**Exon only** (n = 61)	*r* = -0.611 P < 0.001	*r* = -0.516 P < 0.001	*r* = -0.784 P < 0.001	*r* = -0.398 P = 0.002
**Intronic** (n = 14)	*r* = 0.031 P = 0.917	*r* = 0.113 P = 0.699	*r* = 0.124 P = 0.673	*r* = -0.041 P = 0.889

r denotes correlation co-efficiency

### Specific circRNA–miRNA-mRNA regulatory network

As we observed that the changes in the target miRNA binding sites in dysregulated circRNAs is negatively correlated with the change in their target miRNA expressions, we speculated that circRNAs might have an inhibitory effect on the expression of their target miRNAs, and we searched possible specific interactions between the dysregulated circRNA and CI-miRNA. Among the total of 75 potential interactions, 14 showed significant down-regulation of miRNAs with up-regulation of the upstream circRNAs and six showed significant up-regulation of miRNAs with down-regulation of the upstream circRNAs (**Table 2**and **S1 Fig**).

**Table 2:** Differentially regulated circRNA−miRNA interactions.

	FC	*P*	CI-miRNA	FC	*P*	Adjusted *P*
**up-regulated circRNAs with down-regulated CI-miRNAs**						
mmu_circRNA_39485	1.584	0.034	mmu-miR-188-3p	0.003	0.002	0.017
mmu_circRNA_30261	1.825	0.049	mmu-miR-669e-5p	0.189	0.002	0.017
mmu_circRNA_41406	1.771	0.039	mmu-miR-669e-5p	0.189	0.003	0.017
mmu_circRNA_002170	1.659	0.015	mmu-miR-468-5p	0.354	0.009	0.038
mmu_circRNA_36065	1.702	0.024	mmu-miR-670-5p	0.392	0.009	0.039
mmu_circRNA_36074	1.629	0.036	mmu-miR-764-3p	0.531	0.011	0.045
mmu_circRNA_008996	1.510	0.025	mmu-miR-335-5p	0.547	<0.001	0.001
mmu_circRNA_41406	1.771	0.039	mmu-miR-218-5p	0.603	<0.001	<0.001
mmu_circRNA_42102	1.546	0.010	mmu-let-7g-5p	0.617	<0.001	0.001
mmu_circRNA_30668	1.615	0.028	mmu-miR-181b-5p	0.627	0.001	0.009
mmu_circRNA_008996	1.510	0.025	mmu-miR-15a-5p	0.611	0.001	0.008
mmu_circRNA_30668	1.615	0.028	mmu-miR-181d-5p	0.617	0.005	0.024
mmu_circRNA_19995	1.563	0.023	mmu-miR-330-5p	0.633	0.006	0.026
mmu_circRNA_37987	1.825	0.021	mmu-miR-337-3p	0.660	0.002	0.015
**down-regulated circRNAs with up-regulated CI-miRNAs**						
mmu_circRNA_40595	0.645	0.001	mmu-miR-1903	13.438	0.004	0.022
mmu_circRNA_004229	0.647	0.030	mmu-miR-207	5.680	<0.001	0.006
mmu_circRNA_35542	0.658	0.046	mmu-miR-207	5.680	<0.001	0.006
mmu_circRNA_016800	0.646	0.016	mmu-miR-207	5.680	<0.001	0.006
mmu_circRNA_016800	0.646	0.016	mmu-miR-130b-5p	1.641	0.013	0.049
mmu_circRNA_31968	0.431	0.037	mmu-miR-23a-5p	1.335	0.004	0.022

CI-miRNA: circRNA interacting miRNA.

Using the miRNA target gene prediction program, 2,844 mRNAs were predicted as potential targets of the 14 down-regulated miRNA/up-regulated circRNA interactions and 287 mRNAs were identified as potential targets of the six up-regulated miRNA/down-regulated circRNA interactions.

The mRNA microarray expression data (full data in **S3 File**) identified 331 (11.6%) up-regulated CMI-mRNAs of the 14 down-regulated miRNA/up-regulated circRNA interactions and 2 (0.7%) down-regulated CMI-mRNAs of the six up-regulated miRNA/down-regulated circRNA interactions. In total, 333 mRNAs were identified as differentially expressed CMI-mRNAs in the pilocarpine chronic epilepsy model (**S1 Fig,** see **S2 Table** for list).

### Gene ontology and pathway analysis

Gene ontology analysis was performed to evaluate enrichment of the up-regulated CMI-mRNAs in biological processes. Stem cell division, protein K48-linked ubiquitination, regulation of the synaptic vesicle cycle, cerebral cortex cell migration, and stem cell proliferation were the five most highly enriched biological processes (**Table 3**). In the pathway analysis of the differentially expressed CMI-mRNAs, the top five enriched pathways were the mitogen-activated protein kinase (MAPK) signaling pathway, pathways in cancer, the PI3K-Akt signaling pathway, focal adhesion, and the Ras signaling pathway (**Table 3**, full pathway analysis and GO analyses results in **S4 File**). GO and pathway analyses for the down-regulated CMI-mRNAs were not performed due to the low number of the CMI-mRNAs.

**Table 3:** GO biological process and pathway analysis of the up-regulated CMI-mRNAs.

**GO biological process**	**Fold Enrichment**	**P value**
Stem cell division	4.39	0.0059
Protein K48-linked ubiquitination	4.07	0.0041
Regulation of synaptic vesicle cycle	4.03	0.0363
Cerebral cortex cell migration	3.54	0.0302
Stem cell proliferation	3.54	0.0302
**Pathway analysis**	**Genes involved**	**p value**
MAPK signaling pathway	79 (3.4%)	<0.001
Pathways in cancer	100 (4.3%)	<0.001
PI3K-Akt signaling pathway	91 (3.9%)	<0.001
Focal adhesion	61 (2.6%)	<0.001
Ras signaling pathway	65 (2.8%)	<0.001

GO: gene ontology.

### STRING analysis

**Fig 3:**
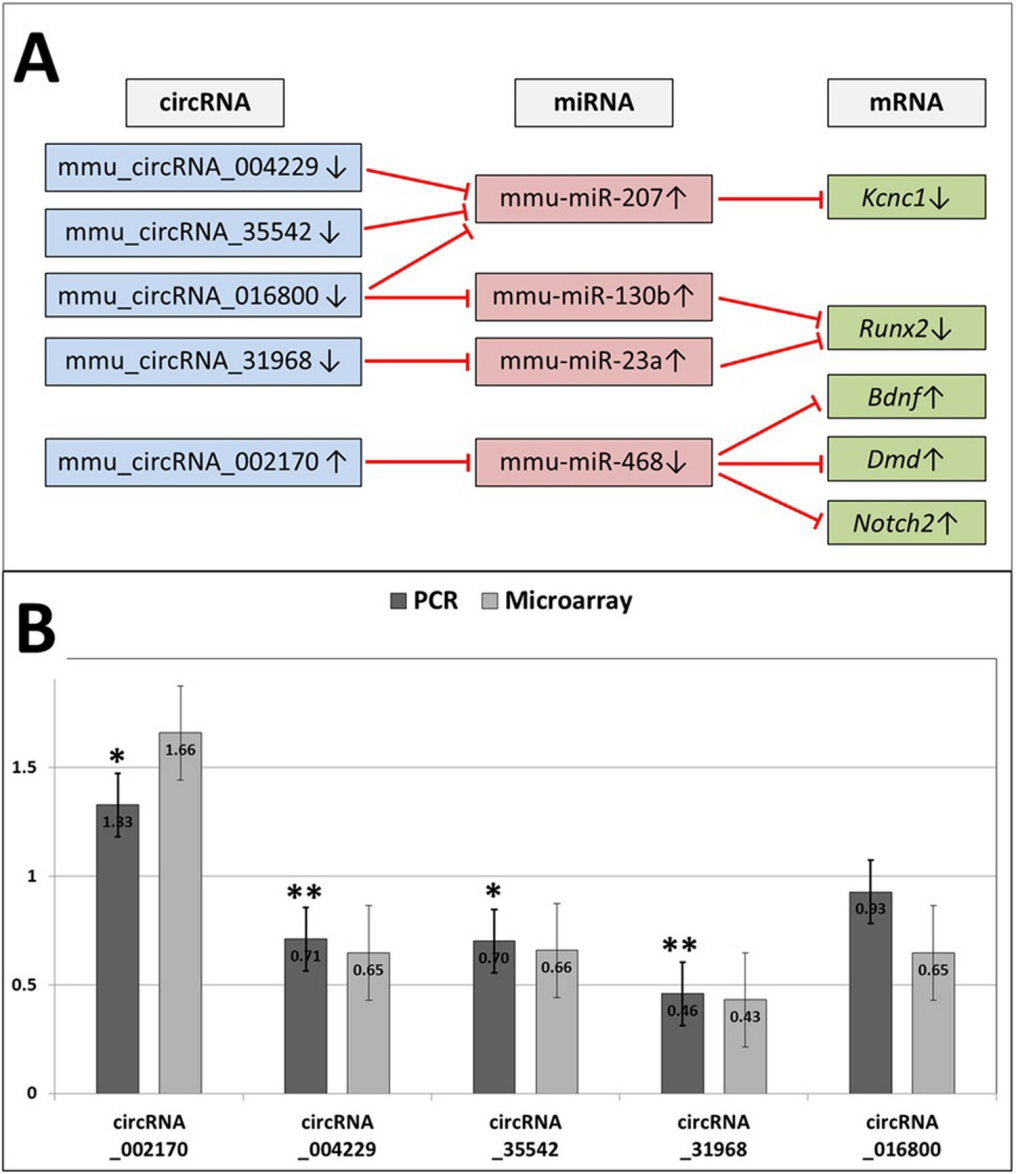
Quantitative PCR validation of differentially expressed circRNAs interacting with pathophysiologically relevant CI-miRNAs and CMI-mRNAs. Some CMI-mRNAs had regulatory interactions with multiple differentially expressed circRNAs, and some circRNAs had interactions with multiple pathophysiologically important CMI-mRNAs. Red bars indicate inhibitory regulation on target miRNAs (panel A). In panel B, expression ratios of the circRNAs (pilocarpine model/control) from the PCR analysis and microarray are shown. *Gapdh* was used as a reference gene to calculate the expression ratios of the circRNAs. CI-mRNA, circRNA-interacting miRNA; CMI-mRNA, circRNA- and miRNA-interacting mRNA. *P < 0.05, **P < 0.01 for the statistical significance of altered expression.

STRING analysis was performed including the 333 CMI-mRNAs. A total of 458 protein–protein interactions were identified and 37 CMI-mRNAs had abundant (≥4) interactions with other differentially expressed CMI-mRNAs (see **Table 4**and **S2 Fig** and **S5 File** for detailed results). Notably, some of these 37 CMI-mRNAs commonly interacted with one upstream CI-miRNA and circRNA, and some CMI-mRNAs also interacted with multiple upstream CI-miRNAs and circRNAs. For example, the up-regulated mRNAs *Bdnf*, *Dmd*, and *Notch2* are commonly regulated by mmu_circRNA_002170, which is also up-regulated, via connection with the down-regulated mmu–miR–468. The down-regulated mRNA *Kcnc1* is multiply regulated by the down-regulated circRNAs mmu_circRNA_016800, 004229, and 35542 via connection with the up-regulated mmu–miR–207 (**Fig 3A**).

**Table 4:** Proteins encoded by the differentially expressed CMI-mRNAs and with abundant protein-protein interactions, from string analysis.

Protein	mRNA fold change	Number of interactions	Protein	mRNA fold change	Number of interactions
Kdr	1.682	24	Tnf	4.858	6
Creb1	1.556	15	Dll4	1.872	6
Fgfr2	1.515	14	Pbx1	1.610	6
Met	1.674	14	Ngf	1.570	6
Fgfr3	1.814	13	Kcnc1	0.580	6
Myod1	1.978	13	Igf1	1.713	6
Mapk4	1.510	13	Bdnf	1.571	6
Lep	5.747	11	Ppara	1.635	6
Notch2	1.598	10	Cd28	1.573	6
Dmd	1.839	10	Wnt1	2.798	5
Hand2	2.409	10	Cul2	1.573	4
Ccr7	1.524	9	Kalrn	1.574	4
Runx2	1.609	9	En2	1.629	4
Crem	1.612	9	Trib1	4.547	4
Itga4	1.638	8	Prdm1	1.700	4
Il6	1.898	7	Tfdp2	1.563	4
Itsn1	1.576	7	Kcnip3	1.589	4
Foxp3	1.598	7	Kcnip1	1.577	4
Maf	1.617	7			

CMI-mRNA: circRNA and miRNA interacting mRNA.

### Quantitative polymerase chain reaction (PCR) validation of circRNA expression

We inferred that the abovementioned circRNAs, which have multiple regulations on the dysregulated CMI-mRNAs with abundant protein-protein interactions, can act as key molecules of epigenetic regulation in chronic epilepsy. PCR analysis of these five differentially expressed circRNAs (mmu_circRNA_002170, 016800, 35542, 004229, and 31968) was performed for validation of the microarray expression data using the primers described in the **S3 Table**. A general consistency was observed between the quantitative PCR and the microarray analysis data. Four out of the five (80.0%) circRNAs (mmu_circRNA_002170, 35542, 004229, and 31968) were confirmed to be differentially expressed in the relevant directions by quantitative PCR analysis (**Fig 3B**). The expression ratio of GAPDH in the hippocampi of pilocarpine/control mice was 1.04 (95% Confidence interval 0.71−1.37, *P* = 0.602, see **S6 File** for the PCR raw data).

## Discussion

This study demonstrated the altered expression of circRNAs and their possible epigenetic regulatory role in the hippocampus of a chronic epilepsy model. The change in the expression of MREs in dysregulated circRNAs had a negative association with their target miRNA expression. This result supports the hypotheses that circRNAs function as miRNA sponges and inhibits the miRNAs via MRE matching.[15, 32] Moreover, MREs of the canonical and the marginal+ full complement categories had a higher negative correlation with the expression of their target miRNA than the incomplete type MREs, indicating that a certain level of nucleotide matching might be required for a circRNA to have a regulatory effect on a target miRNA.[32] However, the altered expression of circRNAs was not associated with a significant regulation of their linear type mRNAs.

Further analyses investigated the possible regulatory network among dysregulated circRNA, miRNA, and mRNA and their pathophysiologic role in chronic epilepsy. Fourty-three dysregulated circRNAs, 20 circRNA−CI-miRNA interactions, and 333 CMI-mRNAs were identified to be possibly involved in the disease-specific circRNA–miRNA-mRNA regulatory networks.

Gene ontology demonstrated that the up-regulated CMI-mRNAs might be closely involved in the pathophysiology of chronic epilepsy. Regarding the biological processes, “stem cell division” and “stem cell proliferation” were among the most enriched terms. These terms might represent the increased proliferation of progenitor cells in the hippocampus, resulting in aberrant neuronal and glial network formation.[1, 2, 6, 36] The enriched term “cerebral cortex cell migration” is related to the dysregulated migration of granule cells in the dentate gyrus of temporal lobe epilepsy.[2, 37] “Protein K48-linked ubiquitination” involves in degradation of disheveled 2 protein, which mediates intracellular transmission of Wnt signals,[38] resulting in the dysregulation of Wnt/β-catenin-mediated neurogenesis and synaptic remodeling.[39] In addition, enriched “regulation of synaptic vesicle cycle” may reflect altered signaling by neurotransmitters such as γ-aminobutyric acid (GABA), adenosine, and glutamate in chronic epilepsy.[40]

For the enriched pathways, the “MAPK signaling” pathway induces proliferation of progenitor cells and differentiation into neurons and astrocytes.[41] The “PI3K-Akt signaling” pathway activates mTOR, which has a crucial role in aberrant neural network formation via enhancement of neural proliferation and synaptogenesis.[42] The “Ras signaling” pathway is an upstream activator of both the MAPK and the PI3K-Akt pathways.[43] Ras also promotes cell proliferation, differentiation, adhesion, migration, and apoptosis, which are also relevant to “pathways in cancer”.[44] “Focal adhesion” if related to the remodeling of neuronal circuits in the hippocampus.[2, 45, 46] Taken together, the CMI-mRNAs are closely related to crucial mechanisms of epilepsy, indicating that circRNAs may have a substantial influence on chronic epilepsy via the circRNA–miRNA–mRNA regulatory networks.

In the STRING analyses, 37 CMI-mRNAs encoded proteins with abundant interactions with the other proteins, suggesting that these genes may have a major pathophysiologic role in chronic epilepsy. Furthermore, some of these CMI-mRNAs had multiple regulatory interactions with circRNAs and some CMI-mRNAs were commonly regulated by one circRNA, implying that these molecules may have a role as a key molecule.[3, 7, 47] For example, the down-regulated *Kcnc1*, which has interactions with six other dysregulated CMI-mRNAs in STING analysis, encodes the KV3.1 subunit of voltage-gated potassium channels. It mediates high-frequency neuronal firing of inhibitory GABAergic interneurons, and its loss-of-function mutation results in a spontaneous seizure.[48] *Kcnc1* is regulated by the multiple down-regulated circRNAs mmu_circRNA_016800, 004229, and 35542 via the up-regulated mmu–miR–207. Runx2 is a transcription factor that is highly expressed in the hippocampus and is involved in cellular homeostasis and glutamate-mediated neuronal excitatory responses.[49, 50] *Runx2* is targeted by upstream down-regulated mmu_circRNA_31968 and up-regulated mmu–miR–23a. The up-regulated mmu_circRNA_002170 interacts with multiple up-regulated CMI-mRNAs *Bdnf*, *Dmd*, and *Notch2* by inhibiting down-regulated mmu–miR–468. *Bdnf* encodes brain-derived natriuretic factor (Bdnf), a neurotrophin that is known to enhance TrkB-mediated aberrant neurogenesis, synaptic formation, and dysregulation of neurotransmitters.[51] *Dmd* encodes dystrophin, which maintains the function of kainate-type glutamate receptors in the hippocampus and affects the susceptibility to seizures.[52] *Notch2* encodes Notch2, which participates in aberrant remodeling of neuronal circuits.[53]

**Fig 4:**
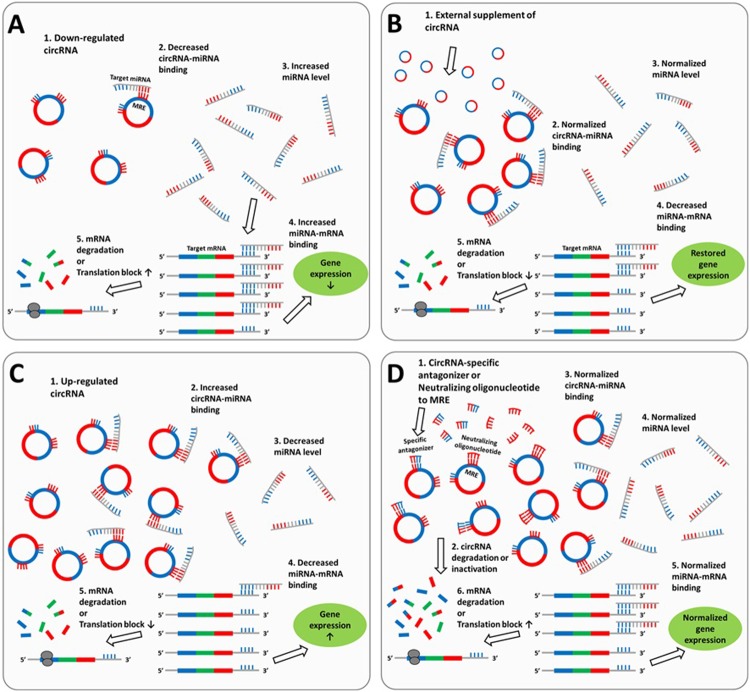
Schematic demonstration of the impact of differentially regulated circRNAs on gene expression and strategies for therapeutic intervention. In panel A, miRNA levels are increased as a result of their reduced sequestration by the down-regulated circRNAs. Binding of miRNAs to their target mRNAs is increased, and the expression levels of the target mRNAs are decreased. By external supplementation of circRNAs, increased sequestration of the target miRNAs results in restoration of the target gene expression (panel B). Panel C describes up-regulated circRNAs excessively inhibiting their target miRNAs, with increased expression of the target genes as a consequence. In this case, external supplementation of oligonucleotides targeting the unique 3'–5' junction sequences of the circRNA or the miRNA response element (MRE) antagonizes the function of the overexpressed circRNAs, resulting in normalization of the target mRNA expression level (panel D).

These circRNAs might serve as potential therapeutic targets as they have multiple regulatory interactions with genes highly involved in the disease pathophysiology. For up-regulated circRNAs, administration of short oligonucleotides that target the unique 3'–5' junction sequences of the circRNA or MREs for the relevant target miRNA might specifically inhibit the function of the circRNA.[47, 54] In contrast, supplementing the down-regulated circRNAs might have an antagomir-like effect on the relevant miRNAs,[20] but with improved stability in the CNS,[9, 11] possibly resulting in the restoration of the target gene function (**Fig 4**).

The present study has some limitations to be addressed. First, only the association between the dysregulated circRNAs, miRNAs, and mRNAs was evaluated, and we did not directly validate the regulatory interactions among them. Second, as the miRNA microarray only partially covered the CI-miRNAs of the dysregulated circRNAs, the complete circRNA−miRNA−mRNA regulatory interactions in chronic epilepsy was not mapped in the current study. Third, correction for the multiple comparisons were not performed for the circRNA microarray analysis, due to a very small portion (43/12,984, 0.33%) of dysregulated circRNAs. However, 4/5 (80.0%) circRNAs which underwent PCR analysis were validated to be relevantly dysregulated. Using a lower *P*-value cutoff (<0.01) might have been an alternative option, but this cutoff value returned no circRNA–miRNA–mRNA regulatory network. Fourth, because of the large number of genes analyzed, validation of the expression ratios with quantitative PCR analyses was not performed for all differentially expressed circRNAs, CI-miRNAs, and CMI-mRNAs. Fifth, the expression of GAPDH, the house-keeping gene for qPCR, varies with inflammatory processes and might not be an ideal house-keeping gene, although the expression of GAPDH in the hippocampi of pilocarpine and control mice in this study were comparable. Future studies should endeavor to confirm the pathophysiologic role of specific circRNA−miRNA−mRNA interactions in chronic epilepsy, as well as other CNS diseases. Furthermore, to enhance the utility of circRNA as a therapeutic target, noninvasive methods of delivering therapeutic molecules into the brain, such as intranasal delivery of circRNA or its antagonists,[20] should be investigated.

## Supporting information

S1 Fig Study flow to demonstrate the interactions of circRNA, miRNA, and mRNA with altered expression in the hippocampus. MRE, miRNA response element, CMI-mRNA, circRNA- and miRNA-interacting mRNA.
(TIF)

S2 Fig STRING analysis of the proteins encoded by the differentially expressed CMI-mRNAs with fold changes of ≥1.5. Visit STRING analysis site (http://string-db.org) for the detailed information of the description of the nodes (proteins) and edges (protein-protein interactions).
(TIF)

S1 Table Differentially expressed circRNAs in the hippocampus of pilocarpine epilepsy model. circRNA: circular RNA and MRE: miRNA response element.
(DOCX)

S2 Table Differentially expressed CMI-mRNAs, with fold changes of ≥1.5. CMI-mRNA: circRNA and miRNA interacting mRNA.
(DOCX)

S3 Table Primers used to validate the five differentially expressed circRNAs. GAPDH was used as a housekeeper gene.
(DOCX)

S1 File Full circular RNA expression profiles at 60 days after status epilepticus. (XLS)

S2 File Full micro RNA expression profiles at 60 days after status epilepticus. (XLS)

S3 File Full mRNA expression profiles at 60 days after status epilepticus. (XLS)

S4 File Pathway analysis and gene ontology analyses for the differentially expressed CMI-mRNAs. (XLSX)

S5 File STRING analysis for the differentially expressed CMI-mRNAs. (XLSX)

S6 File PCR analysis for the differentially expressed circRNAs which have multiple regulations on the dysregulated CMI-mRNAs with abundant protein-protein interactions. (XLSX)
