# Supplementary figures and images for: Possible epigenetic regulatory effect of dysregulated circular RNAs in epilepsy
Source: PLoS One. 2018 Dec 28;13(12):e0209829. doi: 10.1371/journal.pone.0209829 (PMC6310357; doi:10.1371/journal.pone.0209829)

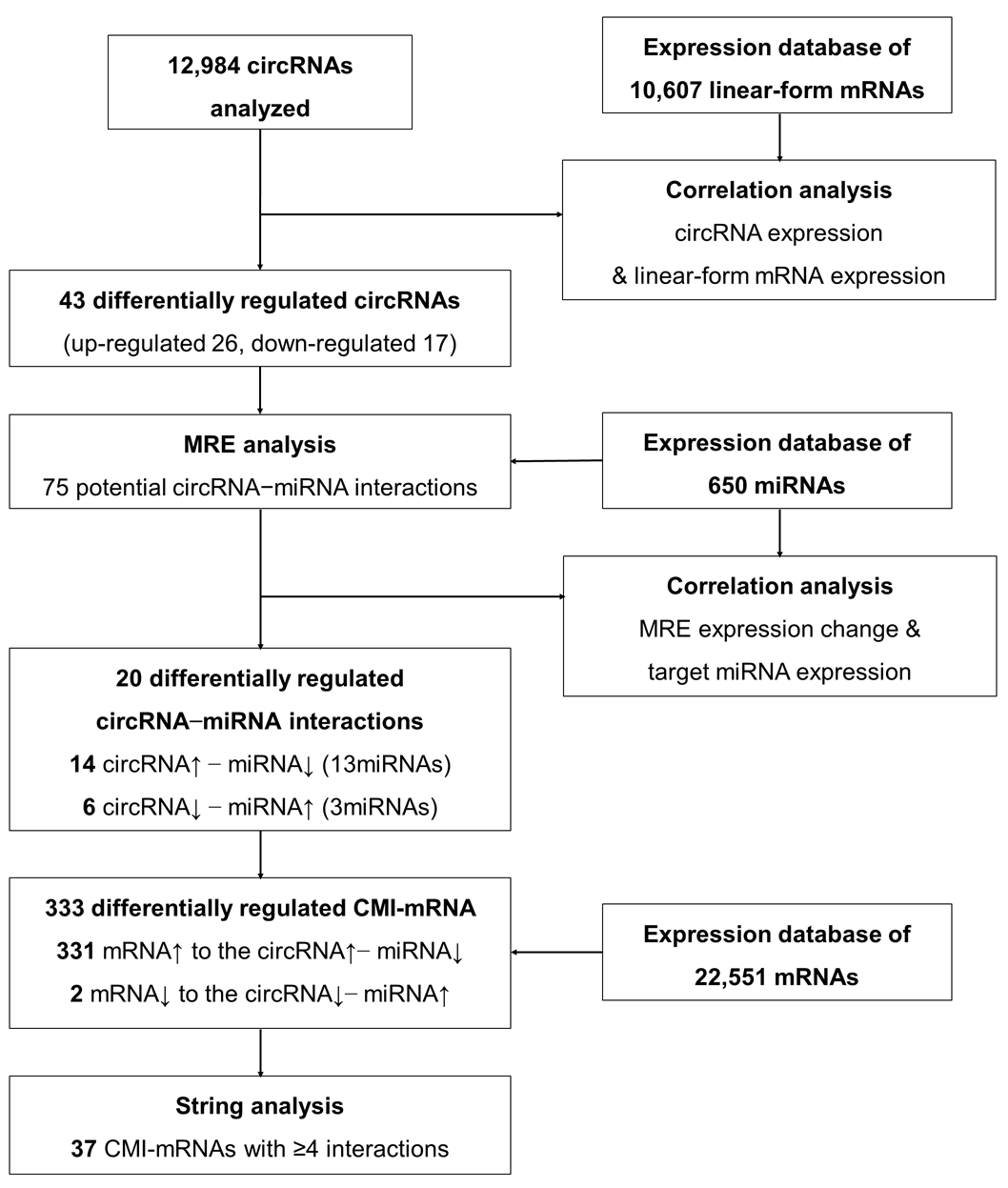

Supplement: S1 Fig — MRE, miRNA response element, CMI-mRNA, circRNA- and miRNA-interacting mRNA. (TIF) [file pone.0209829.s001.tif]

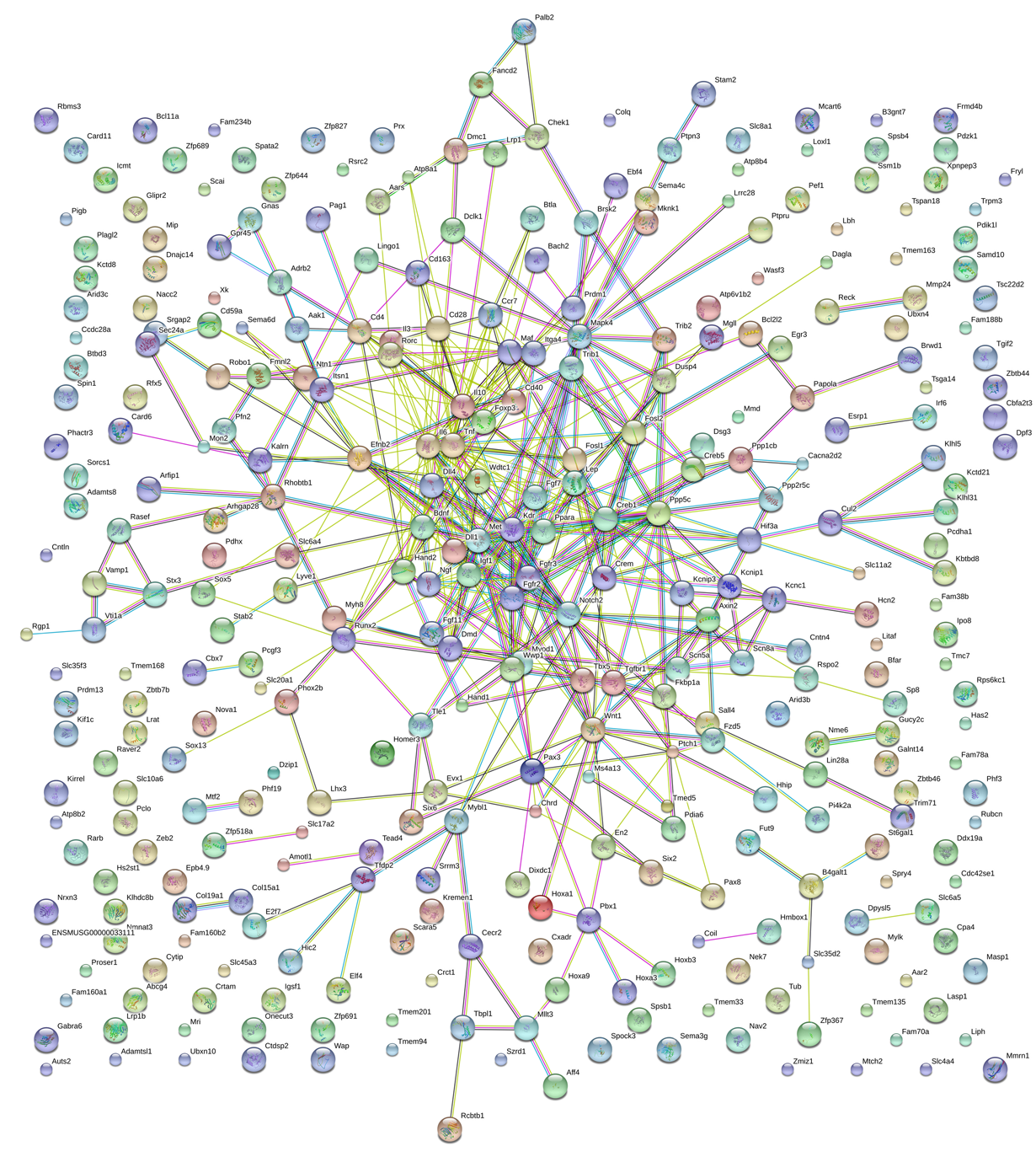

Supplement: S2 Fig — Visit STRING analysis site (http://string-db.org) for the detailed information of the description of the nodes (proteins) and edges (protein-protein interactions). (TIF) [file pone.0209829.s002.tif]
