# Supplementary material for: Possible epigenetic regulatory effect of dysregulated circular RNAs in epilepsy
Source: PLoS One. 2018 Dec 28;13(12):e0209829. doi: 10.1371/journal.pone.0209829 (PMC6310357; doi:10.1371/journal.pone.0209829)
Supplement: S1 Table — circRNA: circular RNA and MRE: miRNA response element. (DOCX) [file pone.0209829.s003.docx]

**S1 Table. Differentially expressed circRNAs in the hippocampus of pilocarpine epilepsy model.**

| **circRNA** | **FC** | **P value** | **type** | **Gene symbol** | **MRE1** | **MRE2** | **MRE3** | **MRE4** | **MRE5** |
| --- | --- | --- | --- | --- | --- | --- | --- | --- | --- |
| **Upregulated** |  |  |  |  |  |  |  |  |  |
| mmu_circRNA_18972 | 2.523 | 0.021 | sense overlapping | AA474331 | mmu-miR-5110 | mmu-miR-1249-5p | mmu-miR-7016-5p | mmu-miR-6981-5p | mmu-miR-149-3p |
| mmu_circRNA_38110 | 2.522 | 0.020 | exonic | *Sema3e* | mmu-miR-5616-5p | mmu-miR-3059-5p | mmu-miR-6962-3p | mmu-miR-7663-3p | mmu-miR-23a-5p |
| mmu_circRNA_30261 | 1.825 | 0.049 | exonic | *Smoc2* | mmu-miR-185-3p | mmu-miR-5120 | mmu-miR-669e-5p | mmu-miR-3100-3p | mmu-miR-7048-5p |
| mmu_circRNA_37987 | 1.825 | 0.021 | exonic | *Chd5* | mmu-miR-712-5p | mmu-miR-337-3p | mmu-miR-320-5p | mmu-miR-7660-5p | mmu-miR-221-5p |
| mmu_circRNA_41406 | 1.771 | 0.039 | exonic | *Prmt3* | mmu-miR-218-5p | mmu-miR-21b | mmu-miR-6984-3p | mmu-miR-6340 | mmu-miR-669e-5p |
| mmu_circRNA_36065 | 1.702 | 0.024 | exonic | 6530403H02Rik | mmu-miR-1967 | mmu-miR-125a-5p | mmu-miR-7214-5p | mmu-miR-670-5p | mmu-miR-6367 |
| mmu_circRNA_32698 | 1.678 | 0.033 | exonic | *Gbf1* | mmu-miR-6342 | mmu-miR-6419 | mmu-miR-6353 | mmu-miR-489-5p | mmu-miR-15b-5p |
| mmu_circRNA_002170 | 1.659 | 0.015 | intergenic |  | mmu-miR-1953 | mmu-miR-6912-5p | mmu-miR-468-5p | mmu-miR-1912-5p | mmu-miR-6378 |
| mmu_circRNA_28575 | 1.655 | 0.042 | intergenic |  | mmu-miR-882 | mmu-miR-199b-5p | mmu-miR-6965-5p | mmu-miR-185-5p | mmu-miR-8100 |
| mmu_circRNA_32678 | 1.630 | 0.039 | exonic | *Mgea5* | mmu-miR-653-3p | mmu-miR-3544-3p | mmu-miR-7230-5p | mmu-miR-23b-5p | mmu-miR-130b-5p |
| mmu_circRNA_36074 | 1.629 | 0.036 | exonic | XLOC_016164 | mmu-miR-7062-5p | mmu-miR-106a-3p | mmu-miR-691 | mmu-miR-764-3p | mmu-miR-8107 |
| mmu_circRNA_28840 | 1.622 | 0.041 | exonic | *Xpnpep3* | mmu-miR-344e-5p | mmu-miR-344h-5p | mmu-miR-29b-2-5p | mmu-miR-669k-5p | mmu-miR-3544-5p |
| mmu_circRNA_42960 | 1.618 | 0.046 | exonic | *Tll1* | mmu-miR-3473c | mmu-miR-3089-5p | mmu-miR-7016-5p | mmu-miR-7037-5p | mmu-miR-207 |
| mmu_circRNA_30668 | 1.615 | 0.028 | exonic | *Adgre1* | mmu-miR-181d-5p | mmu-miR-191-3p | mmu-miR-181b-5p | mmu-miR-7668-3p | mmu-miR-6360 |
| mmu_circRNA_34907 | 1.613 | 0.031 | exonic | *Plcg1* | mmu-miR-7021-3p | mmu-miR-3088-5p | mmu-miR-6999-5p | mmu-miR-3073a-5p | mmu-miR-493-3p |
| mmu_circRNA_45837 | 1.602 | 0.019 | exonic | *Smc1a* | mmu-miR-7026-3p | mmu-miR-29b-2-5p | mmu-miR-107-5p | mmu-miR-6999-3p | mmu-miR-1193-3p |
| mmu_circRNA_008656 | 1.596 | 0.043 | exonic | *Cdk13* | mmu-miR-207 | mmu-miR-7091-3p | mmu-miR-22-5p | mmu-miR-320-5p | mmu-miR-6984-3p |
| mmu_circRNA_39485 | 1.584 | 0.034 | sense overlapping | *Wbscr16* | mmu-miR-466i-3p | mmu-miR-669c-3p | mmu-miR-383-3p | mmu-miR-7041-5p | mmu-miR-188-3p |
| mmu_circRNA_35215 | 1.581 | 0.028 | exonic | *Slc7a14* | mmu-miR-7080-5p | mmu-miR-103-1-5p | mmu-miR-103-2-5p | mmu-miR-7226-5p | mmu-miR-3073b-5p |
| mmu_circRNA_18986 | 1.573 | 0.028 | sense overlapping | *Jmjd1c* | mmu-miR-6946-5p | mmu-miR-7661-5p | mmu-miR-7012-5p | mmu-miR-7027-5p | mmu-miR-7665-5p |
| mmu_circRNA_19995 | 1.563 | 0.023 | sense overlapping | *Pantr1* | mmu-miR-6979-3p | mmu-miR-669n | mmu-miR-330-5p | mmu-miR-7044-5p | mmu-miR-669k-5p |
| mmu_circRNA_38757 | 1.560 | 0.011 | exonic | *Gabrb1* | mmu-miR-7680-5p | mmu-miR-7670-3p | mmu-miR-26a-2-3p | mmu-miR-205-3p | mmu-miR-1954 |
| mmu_circRNA_42102 | 1.546 | 0.009 | exonic | *Ctr9* | mmu-miR-499-3p | mmu-let-7g-5p | mmu-miR-466n-5p | mmu-miR-7223-5p | mmu-miR-653-3p |
| mmu_circRNA_20385 | 1.532 | 0.010 | exonic | *Acsl3* | mmu-miR-29b-2-5p | mmu-miR-7675-3p | mmu-miR-6335 | mmu-miR-7092-3p | mmu-miR-7093-3p |
| mmu_circRNA_008996 | 1.510 | 0.025 | exonic | *Usp34* | mmu-miR-7054-5p | mmu-miR-335-5p | mmu-miR-6999-5p | mmu-miR-7659-3p | mmu-miR-15a-5p |
| mmu_circRNA_34116 | 1.509 | 0.031 | exonic | *Slc1a2* | mmu-miR-141-5p | mmu-miR-3066-5p | mmu-miR-669a-5p | mmu-miR-669p-5p | mmu-miR-3073b-5p |
| **Downregulated** |  |  |  |  |  |  |  |  |  |
| mmu_circRNA_31968 | 0.431 | 0.037 | exonic | *Myo5b* | mmu-miR-23a-5p | mmu-miR-103-1-5p | mmu-miR-103-2-5p | mmu-miR-92a-2-5p | mmu-miR-5615-5p |
| mmu_circRNA_28367 | 0.551 | 0.027 | intergenic |  | mmu-miR-7092-3p | mmu-miR-6344 | mmu-let-7j | mmu-miR-7116-3p | mmu-miR-190a-3p |
| mmu_circRNA_32338 | 0.586 | 0.012 | exonic | *Pip5k1b* | mmu-miR-6982-5p | mmu-miR-6387 | mmu-miR-3068-5p | mmu-miR-6948-3p | mmu-miR-7077-3p |
| mmu_circRNA_30809 | 0.600 | 0.049 | exonic | *L3mbtl4* | mmu-miR-3084-5p | mmu-miR-6769b-5p | mmu-miR-6344 | mmu-miR-7678-3p | mmu-miR-7083-3p |
| mmu_circRNA_017690 | 0.617 | 0.036 | exonic | *Runx1t1* | mmu-miR-7023-5p | mmu-miR-6954-5p | mmu-miR-93-3p | mmu-miR-7117-5p | mmu-miR-7030-5p |
| mmu_circRNA_19057 | 0.627 | 0.035 | sense overlapping | *Pum2* | mmu-miR-7661-5p | mmu-miR-743b-3p | mmu-miR-6339 | mmu-miR-7092-5p | mmu-miR-6984-5p |
| mmu_circRNA_27348 | 0.635 | 0.007 | exonic | *Cacna1d* | mmu-miR-326-5p | mmu-miR-7001-5p | mmu-miR-6953-5p | mmu-miR-6931-5p | mmu-miR-7004-5p |
| mmu_circRNA_43144 | 0.636 | 0.004 | exonic | *Zfp827* | mmu-miR-6919-3p | mmu-miR-7019-3p | mmu-miR-7227-5p | mmu-miR-712-5p | mmu-miR-6961-3p |
| mmu_circRNA_40806 | 0.640 | 0.034 | exonic | *Zfand4* | mmu-miR-7038-3p | mmu-miR-20a-3p | mmu-miR-145a-3p | mmu-miR-346-3p | mmu-miR-149-5p |
| mmu_circRNA_36814 | 0.642 | 0.019 | exonic | *Rad23b* | mmu-miR-1962 | mmu-miR-485-5p | mmu-miR-7085-5p | mmu-miR-762 | mmu-miR-7648-3p |
| mmu_circRNA_40595 | 0.645 | 0.001 | exonic | *Tmf1* | mmu-miR-7116-3p | mmu-miR-6946-3p | mmu-miR-1903 | mmu-miR-295-3p | mmu-miR-148b-5p |
| mmu_circRNA_016800 | 0.646 | 0.016 | exonic | *Khdrbs3* | mmu-miR-130b-5p | mmu-miR-6961-3p | mmu-miR-207 | mmu-miR-7009-3p | mmu-miR-7006-5p |
| mmu_circRNA_004229 | 0.647 | 0.030 | exonic | *Fmn1* | mmu-miR-207 | mmu-miR-7235-3p | mmu-miR-667-5p | mmu-miR-149-5p | mmu-miR-6914-5p |
| mmu_circRNA_21201 | 0.649 | 0.047 | exonic | *Gpr161* | mmu-miR-7032-5p | mmu-miR-128-3p | mmu-miR-361-3p | mmu-miR-6388 | mmu-miR-1843b-5p |
| mmu_circRNA_007217 | 0.656 | 0.035 | sense overlapping | *Hist1h1c* | mmu-miR-7242-5p | mmu-miR-6348 | mmu-miR-208a-5p | mmu-miR-7242-3p |  |
| mmu_circRNA_35542 | 0.658 | 0.046 | exonic | *Gm21949* | mmu-miR-207 | mmu-miR-7094-1-5p | mmu-miR-7042-5p | mmu-miR-92a-2-5p | mmu-miR-7093-3p |
| mmu_circRNA_38483 | 0.663 | 0.005 | exonic | *Htt* | mmu-miR-667-5p | mmu-miR-6899-3p | mmu-miR-8104 | mmu-miR-7011-3p | mmu-miR-6960-5p |

circRNA: circular RNA and MRE: miRNA response element.
