# Supplementary material for: Possible epigenetic regulatory effect of dysregulated circular RNAs in epilepsy
Source: PLoS One. 2018 Dec 28;13(12):e0209829. doi: 10.1371/journal.pone.0209829 (PMC6310357; doi:10.1371/journal.pone.0209829)
Supplement: S2 Table — CMI-mRNA: circRNA and miRNA interacting mRNA. (DOCX) [file pone.0209829.s004.docx]

| **S2 Table. Differentially expressed CMI-mRNAs, with fold changes of ≥1.5.**   \| **Gene Symbol** \| **FC** \| ***P*** \| **Adjusted *P*** \| **EntrezID** \| **Gene Symbol** \| **FC** \| ***P*** \| **Adjusted *P*** \| **EntrezID** \| \| --- \| --- \| --- \| --- \| --- \| --- \| --- \| --- \| --- \| --- \| \| **Up-regulated CMI-mRNAs to the down-regulated miRNAs** \| \| \| \| \| \| \| \| \| \| \| *Lep* \| 5.747 \| 0.000 \| 0.000 \| 16846 \| *Aak1* \| 1.645 \| 0.000 \| 0.001 \| 269774 \| \| *Rbms3* \| 5.648 \| 0.000 \| 0.000 \| 207181 \| *Brwd1* \| 1.64 \| 0.000 \| 0.001 \| 93871 \| \| *Ms4a13* \| 4.999 \| 0.000 \| 0.000 \| 73466 \| *Egr3* \| 1.632 \| 0.000 \| 0.001 \| 13655 \| \| *Tnf* \| 4.858 \| 0.000 \| 0.000 \| 21926 \| *Fancd2* \| 1.628 \| 0.000 \| 0.001 \| 211651 \| \| *Trib1* \| 4.547 \| 0.000 \| 0.000 \| 211770 \| *Arfip1* \| 1.626 \| 0.000 \| 0.001 \| 99889 \| \| *Dsg3* \| 4.21 \| 0.000 \| 0.000 \| 13512 \| *Spsb1* \| 1.624 \| 0.000 \| 0.001 \| 74646 \| \| *Mmrn1* \| 3.893 \| 0.000 \| 0.000 \| 70945 \| *Cd4* \| 1.617 \| 0.000 \| 0.001 \| 12504 \| \| *Lingo1* \| 3.699 \| 0.000 \| 0.000 \| 235402 \| *3830406C13Rik* \| 1.615 \| 0.000 \| 0.001 \| 218734 \| \| *Fam78a* \| 3.373 \| 0.000 \| 0.000 \| 241303 \| *Crem* \| 1.612 \| 0.000 \| 0.001 \| 12916 \| \| *Elf4* \| 2.966 \| 0.000 \| 0.000 \| 56501 \| *Scai* \| 1.611 \| 0.000 \| 0.001 \| 320271 \| \| *Fam188b* \| 2.923 \| 0.000 \| 0.000 \| 330323 \| *Pbx1* \| 1.61 \| 0.000 \| 0.001 \| 18514 \| \| *Hoxa9* \| 2.914 \| 0.000 \| 0.000 \| 15405 \| *D4Ertd22e* \| 1.61 \| 0.000 \| 0.001 \| 213491 \| \| *Cbx7* \| 2.884 \| 0.000 \| 0.000 \| 52609 \| *8430419L09Rik* \| 1.609 \| 0.000 \| 0.001 \| 74525 \| \| *Wnt1* \| 2.798 \| 0.000 \| 0.000 \| 22408 \| *Phactr3* \| 1.609 \| 0.000 \| 0.001 \| 74189 \| \| *Il10* \| 2.749 \| 0.000 \| 0.000 \| 16153 \| *Mtch2* \| 1.605 \| 0.000 \| 0.001 \| 56428 \| \| *Crct1* \| 2.745 \| 0.000 \| 0.000 \| 74175 \| *Fzd5* \| 1.604 \| 0.000 \| 0.001 \| 14367 \| \| *Ebf4* \| 2.663 \| 0.000 \| 0.000 \| 228598 \| *Btla* \| 1.602 \| 0.000 \| 0.001 \| 208154 \| \| *Bcl11a* \| 2.609 \| 0.000 \| 0.000 \| 14025 \| *Sema3g* \| 1.6 \| 0.000 \| 0.001 \| 218877 \| \| *Colq* \| 2.573 \| 0.000 \| 0.000 \| 382864 \| *Notch2* \| 1.598 \| 0.000 \| 0.001 \| 18129 \| \| *Slc4a4* \| 2.561 \| 0.000 \| 0.000 \| 54403 \| *Axin2* \| 1.597 \| 0.000 \| 0.001 \| 12006 \| \| *Slc35d2* \| 2.518 \| 0.000 \| 0.000 \| 70484 \| *Ctdsp2* \| 1.589 \| 0.000 \| 0.001 \| 52468 \| \| *Klhl5* \| 2.51 \| 0.000 \| 0.000 \| 71778 \| *Hic2* \| 1.584 \| 0.000 \| 0.001 \| 58180 \| \| *Zmiz1* \| 2.496 \| 0.000 \| 0.000 \| 328365 \| *Tmem135* \| 1.583 \| 0.000 \| 0.001 \| 72759 \| \| *Sox5* \| 2.487 \| 0.000 \| 0.000 \| 20678 \| *Aff4* \| 1.583 \| 0.000 \| 0.001 \| 93736 \| \| *Zfp367* \| 2.411 \| 0.000 \| 0.000 \| 238673 \| *Nrxn3* \| 1.577 \| 0.000 \| 0.001 \| 18191 \| \| *Trim71* \| 2.355 \| 0.000 \| 0.000 \| 636931 \| *Itsn1* \| 1.576 \| 0.000 \| 0.001 \| 16443 \| \| *Fosl2* \| 2.347 \| 0.000 \| 0.000 \| 14284 \| *Dpysl5* \| 1.575 \| 0.000 \| 0.001 \| 65254 \| \| *Slc6a5* \| 2.343 \| 0.000 \| 0.000 \| 104245 \| *Coil* \| 1.574 \| 0.000 \| 0.001 \| 12812 \| \| *Fmnl2* \| 2.327 \| 0.000 \| 0.000 \| 71409 \| *B4galt1* \| 1.573 \| 0.000 \| 0.001 \| 14595 \| \| *Pax3* \| 2.314 \| 0.000 \| 0.000 \| 18505 \| *Cul2* \| 1.573 \| 0.000 \| 0.001 \| 71745 \| \| *Pcdhac1* \| 2.312 \| 0.000 \| 0.000 \| 353236 \| *Btbd3* \| 1.569 \| 0.000 \| 0.001 \| 228662 \| \| *Chek1* \| 2.31 \| 0.000 \| 0.000 \| 12649 \| *Tfdp2* \| 1.563 \| 0.000 \| 0.001 \| 211586 \| \| *Lrp1* \| 2.264 \| 0.000 \| 0.000 \| 16971 \| *Pef1* \| 1.563 \| 0.000 \| 0.001 \| 67898 \| \| *Mon2* \| 2.22 \| 0.000 \| 0.000 \| 67074 \| *Tub* \| 1.554 \| 0.000 \| 0.001 \| 22141 \| \| *Hif3a* \| 2.216 \| 0.000 \| 0.000 \| 53417 \| *Litaf* \| 1.551 \| 0.000 \| 0.001 \| 56722 \| \| *Fam160b2* \| 2.204 \| 0.000 \| 0.000 \| 239170 \| *Hhip* \| 1.549 \| 0.000 \| 0.001 \| 15245 \| \| *Col15a1* \| 2.199 \| 0.000 \| 0.000 \| 12819 \| *Bfar* \| 1.548 \| 0.000 \| 0.001 \| 67118 \| \| *Fam160a1* \| 2.177 \| 0.000 \| 0.000 \| 229488 \| *Epb4.9* \| 1.546 \| 0.000 \| 0.001 \| 13829 \| \| *Cntn4* \| 2.175 \| 0.000 \| 0.000 \| 269784 \| *Ppp5c* \| 1.545 \| 0.000 \| 0.001 \| 19060 \| \| *Ubxn4* \| 2.165 \| 0.000 \| 0.000 \| 67812 \| *Rspo2* \| 1.542 \| 0.000 \| 0.001 \| 239405 \| \| *Kctd8* \| 2.16 \| 0.000 \| 0.000 \| 243043 \| *Srgap2* \| 1.532 \| 0.000 \| 0.001 \| 14270 \| \| *Nek7* \| 2.1 \| 0.000 \| 0.000 \| 59125 \| *Sorcs1* \| 1.531 \| 0.000 \| 0.001 \| 58178 \| \| *Zfp689* \| 2.087 \| 0.000 \| 0.000 \| 71131 \| *Scn8a* \| 1.53 \| 0.000 \| 0.001 \| 20273 \| \| *Arhgap28* \| 2.06 \| 0.000 \| 0.000 \| 268970 \| *Fut9* \| 1.53 \| 0.000 \| 0.001 \| 14348 \| \| *Nova1* \| 2.044 \| 0.000 \| 0.000 \| 664883 \| *Gucy2c* \| 1.527 \| 0.000 \| 0.001 \| 14917 \| \| *Adrb2* \| 2.034 \| 0.000 \| 0.000 \| 11555 \| *Ccdc28a* \| 1.526 \| 0.000 \| 0.001 \| 215814 \| \| *Scn5a* \| 1.999 \| 0.000 \| 0.000 \| 20271 \| *Dzip1* \| 1.524 \| 0.000 \| 0.001 \| 66573 \| \| *Esrp1* \| 1.99 \| 0.000 \| 0.000 \| 207920 \| *Lbh* \| 1.519 \| 0.000 \| 0.001 \| 77889 \| \| *Hmbox1* \| 1.978 \| 0.000 \| 0.000 \| 219150 \| *Atp6v1b2* \| 1.518 \| 0.000 \| 0.001 \| 11966 \| \| *Myod1* \| 1.978 \| 0.000 \| 0.000 \| 17927 \| *Ppp2r5c* \| 1.517 \| 0.000 \| 0.001 \| 26931 \| \| *Lasp1* \| 1.945 \| 0.000 \| 0.000 \| 16796 \| *Xpnpep3* \| 1.513 \| 0.000 \| 0.001 \| 321003 \| \| *Tle1* \| 1.923 \| 0.000 \| 0.000 \| 21885 \| *Ipo8* \| 1.512 \| 0.000 \| 0.001 \| 320727 \| \| *Pfn2* \| 1.911 \| 0.000 \| 0.000 \| 18645 \| *Dnajc14* \| 1.51 \| 0.000 \| 0.001 \| 74330 \| \| *Slc35f3* \| 1.893 \| 0.000 \| 0.000 \| 210027 \| *Samd10* \| 1.51 \| 0.000 \| 0.001 \| 229011 \| \| *Rarb* \| 1.889 \| 0.000 \| 0.000 \| 218772 \| *Brsk2* \| 1.509 \| 0.000 \| 0.001 \| 75770 \| \| *Atp8b2* \| 1.885 \| 0.000 \| 0.000 \| 54667 \| *Atp8a1* \| 1.509 \| 0.000 \| 0.001 \| 11980 \| \| *Sox13* \| 1.881 \| 0.000 \| 0.000 \| 20668 \| *Kctd21* \| 1.509 \| 0.000 \| 0.001 \| 622320 \| \| *Hand1* \| 1.862 \| 0.000 \| 0.000 \| 15110 \| *Fryl* \| 1.504 \| 0.000 \| 0.001 \| 72313 \| \| *Papola* \| 1.852 \| 0.000 \| 0.000 \| 18789 \| *Sema6d* \| 1.504 \| 0.000 \| 0.001 \| 214968 \| \| *St6gal1* \| 1.838 \| 0.000 \| 0.000 \| 20440 \| *Creb5* \| 1.503 \| 0.000 \| 0.001 \| 231991 \| \| *Spry4* \| 1.828 \| 0.000 \| 0.000 \| 24066 \| *Sp8* \| 1.502 \| 0.000 \| 0.001 \| 320145 \| \| *Card6* \| 1.826 \| 0.000 \| 0.000 \| 239319 \| *Dagla* \| 1.502 \| 0.000 \| 0.001 \| 269060 \| \| *Bcl2l2* \| 1.826 \| 0.000 \| 0.000 \| 12050 \| *Kbtbd8* \| 1.501 \| 0.000 \| 0.001 \| 243574 \| \| *Atp8b4* \| 1.82 \| 0.000 \| 0.000 \| 241633 \| *Spata2* \| 1.501 \| 0.000 \| 0.001 \| 263876 \| \| *Slc45a3* \| 1.818 \| 0.000 \| 0.000 \| 212980 \| *Zbtb7b* \| 1.501 \| 0.000 \| 0.001 \| 22724 \| \| *Evx1* \| 1.808 \| 0.000 \| 0.000 \| 14028 \| *Stab2* \| 2.383 \| 0.000 \| 0.002 \| 192188 \| \| *E2f7* \| 1.805 \| 0.000 \| 0.000 \| 52679 \| *Tead4* \| 2.234 \| 0.000 \| 0.002 \| 21679 \| \| *Vamp1* \| 1.799 \| 0.000 \| 0.000 \| 22317 \| *Dpf3* \| 2.125 \| 0.000 \| 0.002 \| 70127 \| \| *Pdzk1* \| 1.797 \| 0.000 \| 0.000 \| 59020 \| *Zeb2* \| 2.029 \| 0.000 \| 0.002 \| 24136 \| \| *Il3* \| 1.794 \| 0.000 \| 0.000 \| 16187 \| *Dll1* \| 1.824 \| 0.000 \| 0.002 \| 13388 \| \| *Tmem201* \| 1.784 \| 0.000 \| 0.000 \| 230917 \| *Hoxa1* \| 1.751 \| 0.000 \| 0.002 \| 15394 \| \| *Rfx5* \| 1.774 \| 0.000 \| 0.000 \| 53970 \| *Fam38b* \| 1.713 \| 0.000 \| 0.002 \| 667742 \| \| *Icmt* \| 1.763 \| 0.000 \| 0.000 \| 57295 \| *Mtf2* \| 1.706 \| 0.000 \| 0.002 \| 17765 \| \| *Adamts8* \| 1.763 \| 0.000 \| 0.000 \| 30806 \| *Crtam* \| 1.7 \| 0.000 \| 0.002 \| 54698 \| \| *Lin28a* \| 1.741 \| 0.000 \| 0.000 \| 83557 \| *Has2* \| 1.661 \| 0.000 \| 0.002 \| 15117 \| \| *Pi4k2a* \| 1.739 \| 0.000 \| 0.000 \| 84095 \| *Lrrc28* \| 1.573 \| 0.000 \| 0.002 \| 67867 \| \| *Rgp1* \| 1.738 \| 0.000 \| 0.000 \| 242406 \| *Tmed5* \| 1.569 \| 0.000 \| 0.002 \| 73130 \| \| *Galnt14* \| 1.735 \| 0.000 \| 0.000 \| 71685 \| *Zfp518a* \| 1.548 \| 0.000 \| 0.002 \| 72672 \| \| *Zfp644* \| 1.727 \| 0.000 \| 0.000 \| 52397 \| *Tsc22d2* \| 1.545 \| 0.000 \| 0.002 \| 72033 \| \| *Hs2st1* \| 1.72 \| 0.000 \| 0.000 \| 23908 \| *Spin1* \| 1.528 \| 0.000 \| 0.002 \| 20729 \| \| *Lrp1b* \| 1.717 \| 0.000 \| 0.000 \| 94217 \| *Cd40* \| 1.509 \| 0.000 \| 0.002 \| 21939 \| \| *Igf1* \| 1.713 \| 0.000 \| 0.000 \| 16000 \| *Stam2* \| 2.615 \| 0.000 \| 0.003 \| 56324 \| \| *Tmem163* \| 1.702 \| 0.000 \| 0.000 \| 72160 \| *Phox2b* \| 2.588 \| 0.000 \| 0.003 \| 18935 \| \| *Prdm1* \| 1.7 \| 0.000 \| 0.000 \| 12142 \| *Klhl31* \| 2.565 \| 0.000 \| 0.003 \| 244923 \| \| *Zfp691* \| 1.698 \| 0.000 \| 0.000 \| 195522 \| *Phf3* \| 2.071 \| 0.000 \| 0.003 \| 213109 \| \| *Mknk1* \| 1.697 \| 0.000 \| 0.000 \| 17346 \| *Slc17a2* \| 1.994 \| 0.000 \| 0.003 \| 218103 \| \| *Tgfbr1* \| 1.691 \| 0.000 \| 0.000 \| 21812 \| *1700021K19Rik* \| 1.941 \| 0.000 \| 0.003 \| 100502698 \| \| *Phf19* \| 1.691 \| 0.000 \| 0.000 \| 74016 \| *0610011L14Rik* \| 1.932 \| 0.000 \| 0.003 \| 68295 \| \| *Dusp4* \| 1.687 \| 0.000 \| 0.000 \| 319520 \| *Gnas* \| 1.929 \| 0.000 \| 0.003 \| 14683 \| \| *Stx3* \| 1.683 \| 0.000 \| 0.000 \| 20908 \| *Spsb4* \| 1.819 \| 0.000 \| 0.003 \| 211949 \| \| *Kdr* \| 1.682 \| 0.000 \| 0.000 \| 16542 \| *Dixdc1* \| 1.673 \| 0.000 \| 0.003 \| 330938 \| \| *Ptch1* \| 1.68 \| 0.000 \| 0.000 \| 19206 \| *Tsga14* \| 1.657 \| 0.000 \| 0.003 \| 83922 \| \| *Met* \| 1.674 \| 0.000 \| 0.000 \| 17295 \| *Kalrn* \| 1.574 \| 0.000 \| 0.003 \| 545156 \| \| *Nav2* \| 1.672 \| 0.000 \| 0.000 \| 78286 \| *Glipr2* \| 1.561 \| 0.000 \| 0.003 \| 384009 \| \| *Cdc42se1* \| 1.662 \| 0.000 \| 0.000 \| 57912 \| *Hoxb3* \| 1.547 \| 0.000 \| 0.003 \| 15410 \| \| *Irf6* \| 1.656 \| 0.000 \| 0.000 \| 54139 \| *Ptpn3* \| 1.531 \| 0.000 \| 0.003 \| 545622 \| \| *Reck* \| 1.648 \| 0.000 \| 0.000 \| 53614 \| *Ccr7* \| 1.524 \| 0.000 \| 0.003 \| 12775 \| \| *Ppara* \| 1.635 \| 0.000 \| 0.000 \| 19013 \| *Zbtb44* \| 1.519 \| 0.000 \| 0.003 \| 235132 \| \| *Ppp1cb* \| 1.63 \| 0.000 \| 0.000 \| 19046 \| *Chrd* \| 1.508 \| 0.000 \| 0.003 \| 12667 \| \| *En2* \| 1.629 \| 0.000 \| 0.000 \| 13799 \| *2310067B10Rik* \| 1.505 \| 0.000 \| 0.003 \| 71947 \| \| *Slc8a1* \| 1.628 \| 0.000 \| 0.000 \| 20541 \| *Sall4* \| 2.883 \| 0.000 \| 0.004 \| 99377 \| \| *Slc11a2* \| 1.625 \| 0.000 \| 0.000 \| 18174 \| *Rorc* \| 2.373 \| 0.000 \| 0.004 \| 19885 \| \| *Maf* \| 1.617 \| 0.000 \| 0.000 \| 17132 \| *Pax8* \| 1.873 \| 0.000 \| 0.004 \| 18510 \| \| *Fgf7* \| 1.607 \| 0.000 \| 0.000 \| 14178 \| *Arid3c* \| 1.864 \| 0.000 \| 0.004 \| 550619 \| \| *Pdhx* \| 1.604 \| 0.000 \| 0.000 \| 27402 \| *Nmnat3* \| 1.842 \| 0.000 \| 0.004 \| 74080 \| \| *Nacc2* \| 1.6 \| 0.000 \| 0.000 \| 67991 \| *Six6* \| 1.824 \| 0.000 \| 0.004 \| 20476 \| \| *Ddx19a* \| 1.599 \| 0.000 \| 0.000 \| 13680 \| *Cpa4* \| 1.715 \| 0.000 \| 0.004 \| 71791 \| \| *Prdm13* \| 1.597 \| 0.000 \| 0.000 \| 230025 \| *Kirrel* \| 1.678 \| 0.000 \| 0.004 \| 170643 \| \| *Slc6a4* \| 1.591 \| 0.000 \| 0.000 \| 15567 \| *Trib2* \| 1.616 \| 0.000 \| 0.004 \| 217410 \| \| *Cacna2d2* \| 1.587 \| 0.000 \| 0.000 \| 56808 \| *Sec24a* \| 1.533 \| 0.000 \| 0.004 \| 77371 \| \| *Spock3* \| 1.586 \| 0.000 \| 0.000 \| 72902 \| *Slc10a6* \| 2.421 \| 0.000 \| 0.005 \| 75750 \| \| *Fam70a* \| 1.583 \| 0.000 \| 0.000 \| 245386 \| *Pag1* \| 1.97 \| 0.000 \| 0.005 \| 94212 \| \| *Rhobtb1* \| 1.579 \| 0.000 \| 0.000 \| 69288 \| *Kremen1* \| 1.676 \| 0.000 \| 0.005 \| 84035 \| \| *Cd28* \| 1.573 \| 0.000 \| 0.000 \| 12487 \| *Rsrc2* \| 1.616 \| 0.000 \| 0.005 \| 208606 \| \| *Bdnf* \| 1.571 \| 0.000 \| 0.000 \| 12064 \| *Kcnip3* \| 1.589 \| 0.000 \| 0.005 \| 56461 \| \| *Ngf* \| 1.57 \| 0.000 \| 0.000 \| 18049 \| *Abcg4* \| 1.571 \| 0.000 \| 0.005 \| 192663 \| \| *Lyve1* \| 1.567 \| 0.000 \| 0.000 \| 114332 \| *Tmem1nx68* \| 1.534 \| 0.000 \| 0.005 \| 101118 \| \| *Pclo* \| 1.552 \| 0.000 \| 0.000 \| 26875 \| *Mapk4* \| 1.51 \| 0.000 \| 0.005 \| 225724 \| \| *Slc20a1* \| 1.551 \| 0.000 \| 0.000 \| 20515 \| *Tbx5* \| 2.271 \| 0.001 \| 0.006 \| 21388 \| \| *Liph* \| 1.55 \| 0.000 \| 0.000 \| 239759 \| *Il6* \| 1.898 \| 0.001 \| 0.006 \| 16193 \| \| *Auts2* \| 1.549 \| 0.000 \| 0.000 \| 319974 \| *Cd59a* \| 1.807 \| 0.001 \| 0.006 \| 12509 \| \| *Card11* \| 1.548 \| 0.000 \| 0.000 \| 108723 \| *Xk* \| 1.599 \| 0.001 \| 0.006 \| 22439 \| \| *Tmem33* \| 1.528 \| 0.000 \| 0.000 \| 67878 \| *Mcart6* \| 1.53 \| 0.001 \| 0.006 \| 67062 \| \| *Sema4c* \| 1.524 \| 0.000 \| 0.000 \| 20353 \| *Dmc1* \| 1.526 \| 0.001 \| 0.006 \| 13404 \| \| *Wasf3* \| 1.522 \| 0.000 \| 0.000 \| 245880 \| *Cxadr* \| 1.505 \| 0.001 \| 0.006 \| 13052 \| \| *Homer3* \| 1.52 \| 0.000 \| 0.000 \| 26558 \| *Cecr2* \| 2.646 \| 0.001 \| 0.007 \| 330409 \| \| *Fgfr2* \| 1.515 \| 0.000 \| 0.000 \| 14183 \| *Rps6kc1* \| 2.113 \| 0.001 \| 0.007 \| 320119 \| \| *Pdik1l* \| 1.509 \| 0.000 \| 0.000 \| 230809 \| *Gpr45* \| 1.525 \| 0.001 \| 0.007 \| 93690 \| \| *Raver2* \| 1.503 \| 0.000 \| 0.000 \| 242570 \| *Fgfr3* \| 1.814 \| 0.001 \| 0.008 \| 14184 \| \| *Arid3b* \| 3.405 \| 0.000 \| 0.001 \| 56380 \| *Tgif2* \| 1.723 \| 0.001 \| 0.008 \| 228839 \| \| *Aars* \| 3.248 \| 0.000 \| 0.001 \| 234734 \| *2810046L04Rik* \| 1.589 \| 0.001 \| 0.008 \| 212127 \| \| *Pcgf3* \| 3.111 \| 0.000 \| 0.001 \| 69587 \| *Fgf11* \| 1.555 \| 0.001 \| 0.008 \| 14166 \| \| *Mgll* \| 2.956 \| 0.000 \| 0.001 \| 23945 \| *Wap* \| 2.032 \| 0.001 \| 0.01 \| 22373 \| \| *Tmc7* \| 2.446 \| 0.000 \| 0.001 \| 209760 \| *Adamtsl1* \| 1.845 \| 0.001 \| 0.01 \| 77739 \| \| *Pigb* \| 2.444 \| 0.000 \| 0.001 \| 55981 \| *Robo1* \| 1.839 \| 0.001 \| 0.01 \| 19876 \| \| *Pdia6* \| 2.394 \| 0.000 \| 0.001 \| 71853 \| *Dmd* \| 1.839 \| 0.001 \| 0.01 \| 13405 \| \| *Scara5* \| 2.392 \| 0.000 \| 0.001 \| 71145 \| *Onecut3* \| 1.579 \| 0.001 \| 0.01 \| 246086 \| \| *3110062M04Rik* \| 2.363 \| 0.000 \| 0.001 \| 78412 \| *Wdtc1* \| 1.54 \| 0.001 \| 0.01 \| 230796 \| \| *Vti1a* \| 2.19 \| 0.000 \| 0.001 \| 53611 \| *Bach2* \| 1.536 \| 0.001 \| 0.01 \| 12014 \| \| *Fosl1* \| 2.133 \| 0.000 \| 0.001 \| 14283 \| *Mip* \| 2 \| 0.001 \| 0.011 \| 17339 \| \| *Zfp827* \| 2.081 \| 0.000 \| 0.001 \| 622675 \| *Gabra6* \| 1.817 \| 0.001 \| 0.012 \| 14399 \| \| *Ptpru* \| 2.022 \| 0.000 \| 0.001 \| 19273 \| *Kcnip1* \| 1.577 \| 0.001 \| 0.012 \| 70357 \| \| *Trpm3* \| 2.009 \| 0.000 \| 0.001 \| 226025 \| *Efnb2* \| 1.612 \| 0.001 \| 0.013 \| 13642 \| \| *Klhdc8b* \| 1.958 \| 0.000 \| 0.001 \| 78267 \| *B3gnt7* \| 1.578 \| 0.001 \| 0.013 \| 227327 \| \| *Masp1* \| 1.956 \| 0.000 \| 0.001 \| 17174 \| *Creb1* \| 1.556 \| 0.001 \| 0.013 \| 12912 \| \| *Nme6* \| 1.907 \| 0.000 \| 0.001 \| 54369 \| *Ubxn10* \| 1.521 \| 0.001 \| 0.013 \| 212190 \| \| *Dll4* \| 1.872 \| 0.000 \| 0.001 \| 54485 \| *Prx* \| 1.609 \| 0.002 \| 0.015 \| 19153 \| \| *Hoxa3* \| 1.846 \| 0.000 \| 0.001 \| 15400 \| *Ntn1* \| 1.817 \| 0.002 \| 0.016 \| 18208 \| \| *Tbpl1* \| 1.846 \| 0.000 \| 0.001 \| 237336 \| *Tspan18* \| 2.073 \| 0.002 \| 0.017 \| 241556 \| \| *Palb2* \| 1.804 \| 0.000 \| 0.001 \| 233826 \| *Mybl1* \| 1.502 \| 0.002 \| 0.017 \| 17864 \| \| *Cbfa2t3* \| 1.802 \| 0.000 \| 0.001 \| 12398 \| *Cytip* \| 1.618 \| 0.002 \| 0.018 \| 227929 \| \| *Kif1c* \| 1.737 \| 0.000 \| 0.001 \| 16562 \| *Lrat* \| 1.545 \| 0.002 \| 0.019 \| 79235 \| \| *Loxl1* \| 1.731 \| 0.000 \| 0.001 \| 16949 \| *Myh8* \| 1.607 \| 0.003 \| 0.023 \| 17885 \| \| *Amotl1* \| 1.725 \| 0.000 \| 0.001 \| 75723 \| *Igsf1* \| 1.511 \| 0.003 \| 0.024 \| 209268 \| \| *Lhx3* \| 1.702 \| 0.000 \| 0.001 \| 16871 \| *Hand2* \| 2.409 \| 0.003 \| 0.026 \| 15111 \| \| *Mllt3* \| 1.695 \| 0.000 \| 0.001 \| 70122 \| *Col19a1* \| 1.655 \| 0.004 \| 0.032 \| 12823 \| \| *Wwp1* \| 1.693 \| 0.000 \| 0.001 \| 107568 \| *Cntln* \| 1.64 \| 0.004 \| 0.034 \| 338349 \| \| *Zbtb46* \| 1.682 \| 0.000 \| 0.001 \| 72147 \| *2610305D13Rik* \| 1.538 \| 0.004 \| 0.034 \| 112422 \| \| *Frmd4b* \| 1.678 \| 0.000 \| 0.001 \| 232288 \| *Six2* \| 1.883 \| 0.004 \| 0.035 \| 20472 \| \| *Mylk* \| 1.672 \| 0.000 \| 0.001 \| 107589 \| *Plagl2* \| 1.806 \| 0.004 \| 0.035 \| 54711 \| \| *Mmp24* \| 1.672 \| 0.000 \| 0.001 \| 17391 \| *Foxp3* \| 1.598 \| 0.004 \| 0.036 \| 20371 \| \| *Dclk1* \| 1.668 \| 0.000 \| 0.001 \| 13175 \| *Cd163* \| 1.517 \| 0.005 \| 0.04 \| 93671 \| \| *Fkbp1a* \| 1.656 \| 0.000 \| 0.001 \| 14225 \| *Mmd* \| 1.726 \| 0.005 \| 0.043 \| 67468 \| \| *Srrm3* \| 1.655 \| 0.000 \| 0.001 \| 58212 \| *Rasef* \| 1.528 \| 0.005 \| 0.045 \| 242505 \| \| *Hcn2* \| 1.655 \| 0.000 \| 0.001 \| 15166 \| *Itga4* \| 1.638 \| 0.005 \| 0.046 \| 16401 \| \| *Rcbtb1* \| 1.652 \| 0.000 \| 0.001 \| 71330 \|  \|  \|  \|  \|  \| \| **Down-regulated CMI-mRNAs to the up-regulated miRNAs** \| \| \| \| \| \| \| \| \| \| \| *Kcnc1* \| 0.58 \| 0.005 \| 0.046 \| 16502 \| *Runx2* \| 0.638 \| 0.004 \| 0.035 \| 12393 \| |
| --- | --- | --- | --- | --- | --- | --- | --- | --- | --- | --- | --- | --- | --- | --- | --- | --- | --- | --- | --- | --- | --- | --- | --- | --- | --- | --- | --- | --- | --- | --- | --- | --- | --- | --- | --- | --- | --- | --- | --- | --- | --- | --- | --- | --- | --- | --- | --- | --- | --- | --- | --- | --- | --- | --- | --- | --- | --- | --- | --- | --- | --- | --- | --- | --- | --- | --- | --- | --- | --- | --- | --- | --- | --- | --- | --- | --- | --- | --- | --- | --- | --- | --- | --- | --- | --- | --- | --- | --- | --- | --- | --- | --- | --- | --- | --- | --- | --- | --- | --- | --- | --- | --- | --- | --- | --- | --- | --- | --- | --- | --- | --- | --- | --- | --- | --- | --- | --- | --- | --- | --- | --- | --- | --- | --- | --- | --- | --- | --- | --- | --- | --- | --- | --- | --- | --- | --- | --- | --- | --- | --- | --- | --- | --- | --- | --- | --- | --- | --- | --- | --- | --- | --- | --- | --- | --- | --- | --- | --- | --- | --- | --- | --- | --- | --- | --- | --- | --- | --- | --- | --- | --- | --- | --- | --- | --- | --- | --- | --- | --- | --- | --- | --- | --- | --- | --- | --- | --- | --- | --- | --- | --- | --- | --- | --- | --- | --- | --- | --- | --- | --- | --- | --- | --- | --- | --- | --- | --- | --- | --- | --- | --- | --- | --- | --- | --- | --- | --- | --- | --- | --- | --- | --- | --- | --- | --- | --- | --- | --- | --- | --- | --- | --- | --- | --- | --- | --- | --- | --- | --- | --- | --- | --- | --- | --- | --- | --- | --- | --- | --- | --- | --- | --- | --- | --- | --- | --- | --- | --- | --- | --- | --- | --- | --- | --- | --- | --- | --- | --- | --- | --- | --- | --- | --- | --- | --- | --- | --- | --- | --- | --- | --- | --- | --- | --- | --- | --- | --- | --- | --- | --- | --- | --- | --- | --- | --- | --- | --- | --- | --- | --- | --- | --- | --- | --- | --- | --- | --- | --- | --- | --- | --- | --- | --- | --- | --- | --- | --- | --- | --- | --- | --- | --- | --- | --- | --- | --- | --- | --- | --- | --- | --- | --- | --- | --- | --- | --- | --- | --- | --- | --- | --- | --- | --- | --- | --- | --- | --- | --- | --- | --- | --- | --- | --- | --- | --- | --- | --- | --- | --- | --- | --- | --- | --- | --- | --- | --- | --- | --- | --- | --- | --- | --- | --- | --- | --- | --- | --- | --- | --- | --- | --- | --- | --- | --- | --- | --- | --- | --- | --- | --- | --- | --- | --- | --- | --- | --- | --- | --- | --- | --- | --- | --- | --- | --- | --- | --- | --- | --- | --- | --- | --- | --- | --- | --- | --- | --- | --- | --- | --- | --- | --- | --- | --- | --- | --- | --- | --- | --- | --- | --- | --- | --- | --- | --- | --- | --- | --- | --- | --- | --- | --- | --- | --- | --- | --- | --- | --- | --- | --- | --- | --- | --- | --- | --- | --- | --- | --- | --- | --- | --- | --- | --- | --- | --- | --- | --- | --- | --- | --- | --- | --- | --- | --- | --- | --- | --- | --- | --- | --- | --- | --- | --- | --- | --- | --- | --- | --- | --- | --- | --- | --- | --- | --- | --- | --- | --- | --- | --- | --- | --- | --- | --- | --- | --- | --- | --- | --- | --- | --- | --- | --- | --- | --- | --- | --- | --- | --- | --- | --- | --- | --- | --- | --- | --- | --- | --- | --- | --- | --- | --- | --- | --- | --- | --- | --- | --- | --- | --- | --- | --- | --- | --- | --- | --- | --- | --- | --- | --- | --- | --- | --- | --- | --- | --- | --- | --- | --- | --- | --- | --- | --- | --- | --- | --- | --- | --- | --- | --- | --- | --- | --- | --- | --- | --- | --- | --- | --- | --- | --- | --- | --- | --- | --- | --- | --- | --- | --- | --- | --- | --- | --- | --- | --- | --- | --- | --- | --- | --- | --- | --- | --- | --- | --- | --- | --- | --- | --- | --- | --- | --- | --- | --- | --- | --- | --- | --- | --- | --- | --- | --- | --- | --- | --- | --- | --- | --- | --- | --- | --- | --- | --- | --- | --- | --- | --- | --- | --- | --- | --- | --- | --- | --- | --- | --- | --- | --- | --- | --- | --- | --- | --- | --- | --- | --- | --- | --- | --- | --- | --- | --- | --- | --- | --- | --- | --- | --- | --- | --- | --- | --- | --- | --- | --- | --- | --- | --- | --- | --- | --- | --- | --- | --- | --- | --- | --- | --- | --- | --- | --- | --- | --- | --- | --- | --- | --- | --- | --- | --- | --- | --- | --- | --- | --- | --- | --- | --- | --- | --- | --- | --- | --- | --- | --- | --- | --- | --- | --- | --- | --- | --- | --- | --- | --- | --- | --- | --- | --- | --- | --- | --- | --- | --- | --- | --- | --- | --- | --- | --- | --- | --- | --- | --- | --- | --- | --- | --- | --- | --- | --- | --- | --- | --- | --- | --- | --- | --- | --- | --- | --- | --- | --- | --- | --- | --- | --- | --- | --- | --- | --- | --- | --- | --- | --- | --- | --- | --- | --- | --- | --- | --- | --- | --- | --- | --- | --- | --- | --- | --- | --- | --- | --- | --- | --- | --- | --- | --- | --- | --- | --- | --- | --- | --- | --- | --- | --- | --- | --- | --- | --- | --- | --- | --- | --- | --- | --- | --- | --- | --- | --- | --- | --- | --- | --- | --- | --- | --- | --- | --- | --- | --- | --- | --- | --- | --- | --- | --- | --- | --- | --- | --- | --- | --- | --- | --- | --- | --- | --- | --- | --- | --- | --- | --- | --- | --- | --- | --- | --- | --- | --- | --- | --- | --- | --- | --- | --- | --- | --- | --- | --- | --- | --- | --- | --- | --- | --- | --- | --- | --- | --- | --- | --- | --- | --- | --- | --- | --- | --- | --- | --- | --- | --- | --- | --- | --- | --- | --- | --- | --- | --- | --- | --- | --- | --- | --- | --- | --- | --- | --- | --- | --- | --- | --- | --- | --- | --- | --- | --- | --- | --- | --- | --- | --- | --- | --- | --- | --- | --- | --- | --- | --- | --- | --- | --- | --- | --- | --- | --- | --- | --- | --- | --- | --- | --- | --- | --- | --- | --- | --- | --- | --- | --- | --- | --- | --- | --- | --- | --- | --- | --- | --- | --- | --- | --- | --- | --- | --- | --- | --- | --- | --- | --- | --- | --- | --- | --- | --- | --- | --- | --- | --- | --- | --- | --- | --- | --- | --- | --- | --- | --- | --- | --- | --- | --- | --- | --- | --- | --- | --- | --- | --- | --- | --- | --- | --- | --- | --- | --- | --- | --- | --- | --- | --- | --- | --- | --- | --- | --- | --- | --- | --- | --- | --- | --- | --- | --- | --- | --- | --- | --- | --- | --- | --- | --- | --- | --- | --- | --- | --- | --- | --- | --- | --- | --- | --- | --- | --- | --- | --- | --- | --- | --- | --- | --- | --- | --- | --- | --- | --- | --- | --- | --- | --- | --- | --- | --- | --- | --- | --- | --- | --- | --- | --- | --- | --- | --- | --- | --- | --- | --- | --- | --- | --- | --- | --- | --- | --- | --- | --- | --- | --- | --- | --- | --- | --- | --- | --- | --- | --- | --- | --- | --- | --- | --- | --- | --- | --- | --- | --- | --- | --- | --- | --- | --- | --- | --- | --- | --- | --- | --- | --- | --- | --- | --- | --- | --- | --- | --- | --- | --- | --- | --- | --- | --- | --- | --- | --- | --- | --- | --- | --- | --- | --- | --- | --- | --- | --- | --- | --- | --- | --- | --- | --- | --- | --- | --- | --- | --- | --- | --- | --- | --- | --- | --- | --- | --- | --- | --- | --- | --- | --- | --- | --- | --- | --- | --- | --- | --- | --- | --- | --- | --- | --- | --- | --- | --- | --- | --- | --- | --- | --- | --- | --- | --- | --- | --- | --- | --- | --- | --- | --- | --- | --- | --- | --- | --- | --- | --- | --- | --- | --- | --- | --- | --- | --- | --- | --- | --- | --- | --- | --- | --- | --- | --- | --- | --- | --- | --- | --- | --- | --- | --- | --- | --- | --- | --- | --- | --- | --- | --- | --- | --- | --- | --- | --- | --- | --- | --- | --- | --- | --- | --- | --- | --- | --- | --- | --- | --- | --- | --- | --- | --- | --- | --- | --- | --- | --- | --- | --- | --- | --- | --- | --- | --- | --- | --- | --- | --- | --- | --- | --- | --- | --- | --- | --- | --- | --- | --- | --- | --- | --- | --- | --- | --- | --- | --- | --- | --- | --- | --- | --- | --- | --- | --- | --- | --- | --- | --- | --- | --- | --- | --- | --- | --- | --- | --- | --- | --- | --- | --- | --- | --- | --- | --- | --- | --- | --- | --- | --- | --- | --- | --- | --- | --- | --- | --- | --- | --- | --- | --- | --- | --- | --- | --- | --- | --- | --- | --- | --- | --- | --- | --- | --- | --- | --- | --- | --- | --- | --- | --- | --- | --- | --- | --- | --- | --- | --- | --- | --- | --- | --- | --- | --- | --- | --- | --- | --- | --- | --- | --- | --- | --- | --- | --- | --- | --- | --- | --- | --- | --- | --- | --- | --- | --- | --- | --- | --- | --- | --- | --- | --- | --- | --- | --- | --- | --- | --- | --- | --- | --- | --- | --- | --- | --- | --- | --- | --- | --- | --- | --- | --- | --- | --- | --- | --- | --- | --- | --- | --- | --- | --- | --- | --- | --- | --- | --- | --- | --- | --- | --- | --- | --- | --- | --- | --- | --- | --- | --- | --- | --- | --- | --- | --- | --- | --- | --- | --- | --- | --- | --- | --- | --- | --- | --- | --- | --- | --- | --- | --- | --- | --- | --- | --- | --- | --- | --- | --- | --- | --- | --- | --- | --- | --- | --- | --- | --- | --- | --- | --- | --- | --- | --- | --- | --- | --- | --- | --- | --- | --- | --- | --- | --- | --- | --- | --- | --- | --- | --- | --- | --- | --- | --- | --- | --- | --- | --- | --- | --- | --- | --- | --- | --- | --- | --- | --- | --- | --- | --- | --- | --- | --- | --- | --- | --- | --- | --- | --- | --- | --- | --- | --- | --- | --- | --- | --- | --- | --- | --- | --- | --- | --- | --- | --- | --- | --- | --- | --- | --- | --- | --- | --- | --- | --- | --- | --- | --- | --- | --- | --- | --- | --- | --- | --- | --- | --- | --- | --- | --- | --- | --- | --- | --- | --- | --- | --- | --- | --- | --- | --- | --- | --- | --- | --- | --- | --- | --- | --- | --- | --- | --- | --- | --- | --- | --- | --- | --- | --- | --- | --- | --- | --- | --- | --- | --- | --- | --- | --- | --- | --- | --- | --- | --- | --- | --- | --- | --- | --- | --- | --- | --- | --- | --- | --- | --- | --- | --- | --- | --- | --- | --- | --- | --- | --- | --- | --- | --- | --- | --- | --- | --- | --- | --- | --- | --- | --- | --- | --- | --- | --- | --- | --- | --- | --- | --- | --- | --- | --- | --- | --- | --- | --- | --- | --- | --- | --- | --- | --- | --- | --- | --- | --- | --- | --- | --- | --- | --- | --- | --- | --- | --- | --- | --- | --- | --- | --- | --- | --- | --- | --- | --- | --- |

CMI-mRNA: circRNA and miRNA interacting mRNA.
