# Supplementary material for: Possible epigenetic regulatory effect of dysregulated circular RNAs in epilepsy
Source: PLoS One. 2018 Dec 28;13(12):e0209829. doi: 10.1371/journal.pone.0209829 (PMC6310357; doi:10.1371/journal.pone.0209829)
Supplement: S3 Table — GAPDH was used as a housekeeper gene. (DOCX) [file pone.0209829.s005.docx]

**S3 Table. Primers used to validate the five differentially expressed circRNAs.**

| **Gene** | **Primer sequences (5’ to 3’)** | **Size (bp)** | **Annealing (°C)** |
| --- | --- | --- | --- |
| mmu_circRNA_002170 | Forward:5’ CTGGAGCAGCTACTATGGAAA3'  Reverse:5’ TCGGTATCAACGTCCTTAGAA3’ | 55 | 60 |
| mmu_circRNA_ 004229 | Forward:5’ CTCCACCCCAGCACCAGTTG3'  Reverse:5’ TGACAGTCCTCTCCCGCAATG 3’ | 144 | 60 |
| mmu_circRNA_016800 | Forward:5’ CCAACCGGATACAGACCTCC3'  Reverse:5’ CGCTTCAGGGAATTGCCAC 3’ | 111 | 60 |
| mmu_circRNA_31968 | Forward:5’ CAAAACATGGGTGACATGGAC3'  Reverse:5’ CACCTCATCTGGGTCAGGGA 3’ | 107 | 60 |
| mmu_circRNA_35542 | Forward:5’ AGCAGCACACCCGAAGAAC3'  Reverse:5’ GCTGTAGGCTGAGGGGATAGT3’ | 104 | 60 |
| *Gapdh* | Forward:5’ CACTGAGCAAGAGAGGCCCTAT 3'  Reverse:5’ GCAGCGAACTTTATTGATGGTATT 3’ | 136 | 60 |

GAPDH was used as a housekeeper gene.
